# Leukocyte-type 12/15-lipoxygenase is essential for timely inflammation-resolution and effective tissue regeneration following skeletal muscle injury

**DOI:** 10.1101/2025.05.13.653766

**Published:** 2025-05-17

**Authors:** Binayok Sharma, Xinyue Lu, Hamood Rehman, Vandré C. Figueiredo, Carol Davis, Holly Van Remmen, Shihuan Kuang, Susan V. Brooks, Krishna Rao Maddipati, James F. Markworth

**Affiliations:** 1- Department of Animal Sciences, College of Agriculture, Purdue University, West Lafayette, IN, USA; 2- Interdepartmental Nutrition Program (INP), Purdue University, West Lafayette, IN, USA; 3- Center for Aging and the Life Course (CALC), Purdue University, West Lafayette, IN, USA; 4- Department of Biological Sciences, Oakland University, Rochester, MI, USA; 5- Department of Molecular and Integrative Physiology, University of Michigan, Ann Arbor, MI, USA; 6- Aging and Metabolism Research Program, Oklahoma Medical Research Foundation, Oklahoma City, OK, USA; 7- Oklahoma City VA Medical Center, Oklahoma City, OK, USA; 8- Department of Orthopedic Surgery, Duke University School of Medicine, Durham, NC, USA; 9- Department of Pathology, Lipidomic Core Facility, Wayne State University, Detroit, MI, USA; 10- Purdue Institute of Inflammation, Immunology, and Infectious Disease (PI4D), Purdue University, West Lafayette, IN, USA; 11- Indiana Center for Musculoskeletal Health (ICMH), Indiana University, Indianapolis, IN, USA

## Abstract

Unlike traditional anti-inflammatory therapies which may interfere with musculoskeletal tissue repair, pharmacological administration of specialized pro-resolving lipid mediators (SPMs) can promote timely resolution of inflammation while stimulating skeletal muscle regeneration. Despite this, the potential role of endogenous inflammation-resolution circuits in skeletal muscle injury and repair remains unknown. Here, we investigated the effect of whole-body knockout of leukocyte-type 12/15-lipoxygenase (12/15-LOX) on acute inflammation and regeneration following skeletal muscle injury in mice. Prior to muscle injury, *Alox15*^−/−^ mice displayed lower intramuscular concentrations of 12/15-LOX-derived lipid mediators than wild type (WT) mice, and this was associated with chronic low-grade muscle inflammation. *Alox15*^−/−^ mice mounted an exaggerated acute immune response to sterile skeletal muscle injury which was associated with a local imbalance of pro-inflammatory vs. pro-resolving lipid mediators. During the regenerative phase, *Alox15*^−/−^ mice displayed defects in myogenic gene expression, myofiber size, and myonuclear accretion. Mechanistically, bone marrow-derived macrophages (Mϕ) obtained from *Alox15*^−/−^ mice produced less 12/15-LOX-derived lipid mediators and this was associated with impaired M2 polarization. Isolated myogenic progenitor cells also produced many LOX metabolites in response to long chain polyunsaturated fatty acid (LC-PUFA) supplementation, including bioactive SPMs. *Alox15*^−/−^ myoblasts were both impaired in their ability to produce SPMs and were insensitive to the stimulatory effect of LC-PUFAs on *in vitro* myogenesis. These data show that the 12/15-LOX pathway is essential for timely resolution of acute inflammation and direct determination of myogenic progenitor cell fate following skeletal muscle injury.

## Introduction:

Skeletal muscle injury is well-known to induce a robust acute inflammatory response which results in the local appearance of substantial numbers of blood leukocytes^1^. Polymorphonuclear cells (PMNs) (e.g., neutrophils) are among the first blood immune cells to arrive at the site of skeletal muscle injury, usually within minutes to hours^2,3^. The next major type of blood cells that migrate to the site of injury are monocytes that differentiate locally to become tissue macrophages (Mϕs) which play a crucial role in supporting subsequent myofiber regeneration via immune-muscle cell cross-talk^4–6^.

Lipoxygenase (LOX) enzymes are pivotal in modulating inflammation by oxidizing long chain polyunsaturated fatty acids (LC-PUFAs) to form bioactive lipid mediators^7^. In mammals, specific enzymes are responsible for catalyzing enzymatic oxidation of three sites on arachidonic acid (ARA, 20:4n-6), and thus are named 5-, 12-, and 15-LOX, respectively^8^. 15-LOX oxygenates ARA substrate at carbon-15 to form 15-hydroperoxy-eicosatetranoic acid (15-HpETE), which is further reduced to 15-hydroxy-eicosatetranoic acid (15-HETE)^9^. However, certain LOX enzymes possess dual specificities, resulting in differences among species^8^. Importantly, while the human protein encoded by the *ALOX15* gene (15-LOX-1) produces mainly 15-HETE, the analogous protein encoded by the murine *Alox15* gene produces both 12-HETE and 15-HETE and is thus often termed 12/15-LOX.

LOX-derived lipid mediators are involved in a myriad of physiological and pathological processes, including the initiation and resolution of the inflammatory response^10^. Monohydroxylated PUFA metabolites such as 5-, 12-, and 15-HETE may exert direct biological effects^11^. In addition, certain monohydroxy-PUFAs are potential precursors in the subsequent downstream biosynthesis of a complex array of potential downstream di- and tri-hydroxylated PUFA metabolites^12^. For example, 15-HETE formed via 15-LOX metabolism of ARA can be further metabolized via the 5-LOX pathway to form the lipoxin family of lipid mediators (e.g., LXA_4_)^13^. Unlike classical eicosanoids (e.g., prostaglandins and leukotrienes), the lipoxins have potent anti-inflammatory actions such as limiting PMN chemotaxis and degranulation^14^. The lipoxins are also key molecules acting in the resolution phase of the acute inflammatory response by promoting monocyte recruitment to the site of inflammation^15^ and stimulating Mϕ mediated phagocytosis of apoptotic PMNs (efferocytosis)^16^.

LC omega-3 (n-3) PUFAs such as eicosapentaenoic acid (EPA, 20:5n-3), docosapentaenoic acid (DPA, 22:5n-3) and docosahexaenoic acid (DHA, 22:6n-3) are also potential substrates for mammalian LOX enzymes. 15-LOX activity converts DHA to 17-hydroxy-docosahexaenoic acid (17-HDoHE)^17^. Subsequently 5-LOX expressing cells (e.g., PMNs) may transform 17-HDoHE to downstream products such as the D-series resolvins (e.g., RvD1)^18^. Similarly, enzymatic metabolism of n-3 DHA by 12-LOX produces 14-hydroxy-docosahexaenoic acid (14-HDoHE) which can be further converted to the maresins (e.g., MaR1)^19^. Based on their key roles in actively bringing about the resolution phase of the acute inflammatory response the lipoxins, resolvins, protectins, and maresins have collectively been termed specialized pro-resolving lipid mediators (SPMs)^20^.

Recent evidence demonstrated by us and independent groups have used liquid chromatography tandem mass spectrometry (LC-MS/MS) profiling to show that many potential LOX pathway metabolites increase locally following acute skeletal muscle injury^21–26^. These novel findings are consistent with earlier reports showing that exercise-induced skeletal muscle damage also transiently increased many LOX metabolites in human blood^27,28^. Despite their clear association with muscle injury and repair processes, the precise role of such LOX-derived lipid mediators in adaptive skeletal muscle remodeling remains uncertain. In the only published study employing a loss-of-function model to date, leukocyte type 12/15-LOX knockout (*Alox15*^−/−^) mice were found to be protected against skeletal muscle wasting induced by denervation surgery^29^. Nevertheless, no prior study has tested the potential impact of loss of 12/15-LOX activity on acute skeletal muscle injury and ensuing myofiber regeneration.

In the current study we investigated the functional role of the *Alox15* gene, which encodes the murine leukocyte-type 12/15-LOX enzyme, on acute skeletal muscle inflammation and regeneration in mouse and cell models. We hypothesized that 12/15-LOX-mediated enzymatic conversion of LC-PUFAs to form bioactive lipid mediator metabolites with anti-inflammatory, pro-resolving, and tissue reparative actions would be essential for timely resolution of the acute innate immune response and robust skeletal muscle regeneration. Our data reveals not only a crucial role of *Alox15* in the successful resolution of acute skeletal muscle inflammation following sterile tissue damage, but also an unexpectedly direct and indispensable role of 12/15-LOX enzyme activity as a key positive determinant of resident myogenic progenitor cell fate within injured muscle.

## Materials and Methods:

### Animals:

Wild type (WT) C57BL/6J (Jackson, 000664) and whole-body 12/15-LOX knockout (*Alox15*^−/−^) mice on a C57BL/6J background (B6.129S2-*Alox15*^tm1Fun^/J (Jackson, 002778^30^) were sourced from the Jackson Laboratory. Male 6–8-month-old *Alox15*^−/−^ mice and age/sex matched WT mice were used in the current studies. Mice were maintained in a specific pathogen-free (SPF) environment with ad libitum access to food and water.

### Muscle injury:

Mice were anesthetized using 2% isoflurane and muscle injury was induced by bilateral intramuscular injections of 50 μL of 1.2% barium chloride (BaCl_2_) prepared in sterile saline into the tibialis anterior (TA) muscle. Following the procedure, the mice were placed back in their respective cages and closely monitored until ambulatory. To assess the extent of inflammation and regeneration, TA muscles were collected on day 3, day 5, and day 14 following muscle injury.

### Muscle tissue collection:

TA muscles were rapidly dissected under isoflurane anesthesia and muscle weights were recorded. The left TA was snap-frozen in liquid nitrogen for lipidomic analysis. The proximal portion of the right TA was snap frozen in liquid nitrogen for molecular analysis. The distal portion of the right TA was orientated longitudinal on plastic support, covered with a layer of optimal cutting temperature (OCT) compound, and then flash-frozen in isopentane chilled with liquid nitrogen. Samples were stored at −80°C until further analysis.

### Histology and immunofluorescence staining:

Tissue cross-sections (10 μm) were cut from the mid-belly region of OCT embedded TA muscle using a Leica CM1950 cryostat at −20°C. Muscle tissue sections were collected onto SuperFrost Plus slides and air-dried at room temperature. Unfixed sections were used for hematoxylin and eosin (H&E) and muscle fiber type staining. Slides were fixed in acetone for 10 minutes at −20°C before air-drying in preparation for immune cell staining. Prepared slides were blocked using either 10% normal goat serum (GS) (Invitrogen, 10000C) in phosphate buffered saline (PBS) or with Mouse on Mouse (M.O.M.) IgG Blocking Reagent (Vector Laboratories, MKB22131) when mouse primary antibodies were used on mouse tissue samples. The slides were then incubated with primary antibodies prepared in either 10% GS in PBS or M.O.M protein diluent at 4°C overnight. On the following day, the sections were washed in PBS and then incubated with Alexa Fluor-conjugated secondary antibodies (diluted 1:500 in PBS) for 1 h at room temperature. Slides were washed in PBS and then mounted with coverslips using MOWIOL Fluorescence Mounting Medium. Stitched panoramic brightfield and fluorescent images of the entire TA muscle cross section were captured using an automated fluorescent microscope (Echo Revolution) operating in upright configuration. Myofiber morphology, central nuclei fiber identification, and muscle fiber type profile was analyzed by high-throughput full automated image analysis using the MuscleJ 1.0.2 plugin for FIJI/ImageJ^31^.

### Immunohistochemistry antibodies:

Primary antibodies used include MyHC type I [Developmental Studies Hybridoma Bank (DSHB), BA-D5c, 1:100], MyHC type IIA (DSHB, SC-71c, 1:100), MyHC type IIB (DSHB, BF-F3c, 1:100), eMHC (DSHB, F1.652s, 1:20), Ly6G (GR1) (Bio-Rad, MCA2387, 1:50), CD68 (Bio-Rad, MCA1957, 1:200), CD163 (Santa Cruz, sc-58965, 1:200), and laminin (Abcam, ab7463, 1:200). Primary antibody staining was visualized with appropriate Alexa Fluor conjugated secondary antibodies (Invitrogen, 1:500 in PBS). In specific experiments fluorescent dyes including DAPI (Invitrogen, Thermo Fisher Scientific, D21490, 2 μg/mL), wheat germ agglutinin (WGA) Alexa Fluor 350 conjugate (Invitrogen, W11263, 100 μg/mL), and phalloidin (Invitrogen, Thermo Fisher Scientific ActinRed 555 ReadyProbes, R37112) were used to counterstain cell nuclei, extracellular matrix, and muscle fibers, respectively.

### C2C12 Cell culture:

Murine C2C12 myoblasts were sourced from the American Type Culture Collection (ATCC, CRL-1772). Myoblasts were growth at 37°C and 5% CO_2_ in high-glucose Dulbecco’s Modified Eagle’s Medium (DMEM) (Gibco, 11995073), supplemented with 10% fetal bovine serum (FBS) (Corning, 35015CV), and antibiotics [penicillin (100 U/ml) and streptomycin (100 μg/mL)] (Gibco, 15140122). Confluent myoblasts were switched to DMEM supplemented with antibiotics and 2% horse serum (HS) (Gibco, 26050088) to induce myogenic differentiation. To assess their impact on C2C12 myoblast differentiation commercially available LOX inhibitors were prepared in differentiation media and added to confluent myotube cultures at the onset of myogenic differentiation. Drugs tested included: (1) The pan LOX inhibitors 5,8,11,14-Eicosatetraynoic Acid (ETYA) (Cayman Chemicals, 90120), nordihydroguaiaretic acid (NDGA) (Cayman Chemicals, 70300), and baicalein (Cayman Chemicals, 70610). (2) The 15-LOX specific inhibitors BLX3887 (Cayman Chemicals, 27391), 9c(i472) (Cayman Chemicals, 28225), PD 146176 (Cayman Chemicals, 10010518), ML-351 (Cayman Chemicals, 16119), ThioLox (Cayman Chemicals, 38946). (3) The 5-LOX specific inhibitors malotilate (Cayman Chemicals, 30266), zileuton (Cayman Chemicals, 10006967), and MK-886 (Cayman Chemicals, 10133). (4) The 12-LOX specific inhibitors CAY10698 (Cayman Chemicals, 18582) and ML-355 (Cayman Chemicals, 18537). Following 72 h of myogenic differentiation myotube cultures were fixed in 4% paraformaldehyde (PFA) in preparation for immunocytochemistry analysis.

### Primary myoblast culture:

Primary myoblasts were isolated from pooled hindlimb muscles of 6-8 month old male *Alox15*^−/−^ mice utilizing the protocol described by Hindi et al., 2017^32^. Primary myoblasts were cultivated at 37°C and 5% CO_2_ on T75 flasks coated with 10% Matrigel Matrix (Corning, 354234) in growth media (GM) consisting of Ham’s F-10 Nutrient Mixture (Gibco, 11550043), supplemented with 20% FBS, fibroblast growth factor basic (bFGF, 10 ng/mL) (PeproTech, 100-18B), and antibiotics [penicillin (100 U/ml) and streptomycin (100 μg/mL)]. To assess proliferation rate 2 × 10^5^ myoblast were plated per well of Matrigel coated 12-well plates (Thermo Scientific, 130185) and allowed to proliferate in GM for 48 h. To assess myogenic differentiation, 2 × 10^5^ myoblasts were plated per well of Matrigel coated 12-well plates and allowed to proliferate in GM for 72 h before switching to differentiation media (DM) consisting of DMEM supplemented with antibiotics and 2% horse serum (HS) (Gibco, 26050088) for a further duration of 72 h. To test the effect of LC-PUFA supplementation on myotube formation, differentiation media was supplemented with a 25 μM dose of ARA (Cayman Chemicals, 90010), EPA (Cayman Chemicals, 90110), DPA (Cayman Chemicals, 90165), DHA (Cayman Chemicals, 90310), or equimolar mixture of these four fatty acids (6.25 μM each). Following 72 h of myogenic differentiation conditioned cell culture media was collected for analysis of extracellular lipid mediator concentration by LC-MS/MS based metabolipidomic profiling and myotubes were fixed in 4% PFA for immunocytochemistry analysis.

### Immunocytochemistry and image analysis:

To assess cellular morphology, myotubes were fixed in 4% PFA, permeabilized with 0.1% Triton X-100, and blocked in 1% bovine serum albumin (BSA) for 1 h at room temperature. Cells were then incubated overnight at 4°C with blocking buffer containing primary antibodies against sarcomeric MyHC (DSHB, MF20c, 1:100) and myogenin (DSHB, F5Dc, 1:100). The following morning, cells were incubated with secondary antibodies including Goat Anti-Mouse IgG2b Alexa Fluor 647 conjugate (Invitrogen, A-21242, 1:500) and Goat Anti-mouse IgG1 Alexa Fluor 555 conjugate (Invitrogen, A-21127, 1:500) for 1 h at room temperature. DAPI (Invitrogen) D21490, 2 μg/mL was used to counterstain the cell nuclei. Stained cells were visualized with an automated fluorescent microscope (Echo Revolution) operating in inverted configuration. Fluorescent images were automatically captured from a total of 9 predetermined fields of view (FOVs) per well of 12-well culture plates using a 10 × Plan Fluorite objective. To assess average myotube diameter, 50 myotubes per well were analyzed as previously described by us^33^. From each well, 5 FOVs were randomly selected, and the diameters of the 10 largest sarcomeric myosin-positive multinucleated cells in each field were manually measured at their widest uniform point using ImageJ software. For branching myocytes, each branch was measured as a separate myotube, and the region where the branches converge was exclude. To assess total myotube area per FOV, a custom in-house ImageJ macro was employed. Myosin^+^ cell area was automatically quantified for nine FOVs per culture well from 3-4 independent culture wells per experimental group.

### RNA extraction, cDNA synthesis, and RT q-PCR:

Frozen TA muscle samples (10-20 mg) were homogenized for 45 seconds (4 m/s) in 600 μL of ice-cold Trizol reagent (Invitrogen, 15596018) using a Fisherbrand Bead Mill 4 mini homogenizer (Thermo Fisher Scientific, 15-340-164). RNA was extracted from muscle tissue homogenates or cellular lysates by phenol-chloroform phase separation and isopropanol precipitation following the Trizol protocol with minor modifications. The concentration and quality of RNA were assessed by spectrophotometry using a NanoDrop spectrophotometer. To eliminate any contaminating genomic DNA, the extracted RNA was treated with DNase I (Thermo Fisher Scientific, AM2222) which was then deactivated via heat treatment. RNA (1 μg) was reverse transcribed to cDNA using the High-Capacity RNA-to-cDNA Kit (Applied Biosystems, 4387406). Quantitative real-time PCR (RT-qPCR) was carried out on the cDNA samples using a QuantStudio 5 Real-Time PCR System (384-well) (Applied Biosystems, A28570). cDNA samples (1-40 ng dependent on gene of interest) were analyzed in duplicate 10 μL reaction volumes of PowerUp^™^ SYBR^™^ Green Master Mix (Applied Biosystems, A25742) with 1 μM forward and reverse primers. The relative expression levels of mRNA were evaluated using the 2^−ΔΔCT^ method. Skeletal muscle tissue, bone marrow derived Mϕ, and primary myoblast expression of genes of interest were normalized to *Gapdh, Tbp*, and *Rplp0* as internal controls, respectively. Primer sequences are listed in Table 1.

### Protein extraction and western blotting:

Frozen TA muscle samples (10-20 mg) were homogenized for 60 seconds (5 m/s) using a Fisherbrand Bead Mill 4 mini homogenizer (Thermo Fisher Scientific, 15-340-164) in ice-cold 1 × RIPA lysis buffer (MilliporeSigma, 20188) (15 μL/mg tissue) supplemented with a 1 × concentration of Halt Protease and phosphatase inhibitor cocktail (Thermo Fisher Scientific, 78442). The resulting homogenate was agitated at 4°C for 1 h and then clarified by centrifugation at 13,000 g for 10 minutes at 4°C. The supernatant was collected, and protein concentration was determined using a Pierce bicinchoninic acid (BCA) protein assay kit (Thermo Fisher Scientific, PI23225). Protein samples were diluted to a standard concentration in 1 × Laemmli buffer and boiled for 5 min. An equal volume of protein (20 μg) was then separated by sodium dodecyl sulfate-polyacrylamide gel electrophoresis (SDS-PAGE). Proteins were transferred to a nitrocellulose membrane using a Trans-Blot Turbo Transfer System (Bio-Rad, 1704150). Membranes were blocked for 1 h a room temperature in 5% skim milk power in Tris buffered saline with 0.1% Tween 20 (TBST). Membranes were subsequently incubated overnight at 4°C with gentle agitation with a Rabbit Anti-15-LOX-1 primary antibody (Abcam, ab244205) diluted 1:1000 in 5% Bovine Serum Albumin (BSA) in TBST. The following day membranes were washed in TBST and probed with a Goat Anti-Rabbit IgG (H + L) Horseradish Peroxidase (HRP) conjugated secondary antibodies (Jackson Labs, 111-035-144) diluted in 5% skim milk in TBST for 1 h at room temperature. A primary antibody against GAPDH (Santa Cruz, 32233) was used as a loading control which was detected with a Goat Anti-Mouse IgG (H + L) Horseradish Peroxidase (HRP) conjugated secondary antibodies (Jackson Labs, 115-035-146). Protein bands were visualized using Clarity Western enhanced chemiluminescent (ECL) substrate (Bio-Rad, 1705060). Chemiluminescent signals were captured using a ChemiDoc Imaging System (Bio-Rad, 12003153). Densitometry analysis was performed using Image Lab 6.1 software (Bio-Rad).

### ELISA:

Conditioned cell culture media samples (cell culture supernatants) were collected from proliferating myoblasts or differentiating myotubes obtained from WT and *Alox15*^−/−^ mice. Conditioned media samples were centrifuged at 500 × g at 4°C, the supernatant collected, and stored at −80°C. Cell culture media samples were thawed once and analyzed by commercially available ELISA kit for determination of 15(S)-HETE concentration as per the manufactures’ recommendation (Cayman Chemical, 534721).

### Bone marrow-derived Mϕ culture:

Bone marrow cells were collected from the tibias and femurs of WT and *Alox15*^−/−^ mice and cultured for 7 days at 37°C and 5% CO_2_ in Mϕ growth media consisting of high glucose DMEM supplemented with 10% FBS, antibiotics [penicillin (100 U/ml) and streptomycin (100 μg/mL)], and 20 ng/mL macrophage colony stimulating factor (M-CSF) (BioLegend, 576404). The resulting adherent bone marrow-derived Mϕ (BMMs) were then plated in Mϕ growth media supplemented with M-CSF at a density of 2.5 × 10^5^ cells per well of a 12-well plate (for immunocytochemistry) or 5 × 10^5^ cells per well of a 12-well plate (for RNA extraction). Mϕ were allowed to adhere to the plastic surface for 24 h. M-CSF was then withdrawn and BMMs were either maintained as naïve M0 Mϕ in serum free DMEM, polarized to M1 Mϕ by stimulation with 100 ng/mL LPS (Sigma-Aldrich, L2630) and 20 ng/mL interferon gamma (INF-γ) (R&D Systems, 485-MI), or polarized to M2 Mϕ by stimulation with 20 ng/mL interleukin-4 (IL-4) (R&D Systems, 404-ML). The resulting M0, M1, and M2 Mϕ were collected for RNA extraction by lysis in Trizol reagent following 24 h of polarization or fixed in 4% PFA for immunocytochemistry analysis following 48 h of polarization. Conditioned cell culture media was also collected following 24 h polarization for analysis of extracellular lipid mediators by LC-MS/MS based metabolipidomic profiling.

### LC-MS/MS–based metabolipidomic profiling of muscle tissue:

TA muscle samples (20-40 mg) were mechanically homogenized in 1 mL PBS using a bead mill. The tissue homogenates were centrifuged at 3000 × g for 5 minutes and the supernatant was collected. Sample supernatants (0.85 mL) were spiked with 5 ng each of 15(S)-HETE-d8, 14(15)-EpETrE-d8, Resolvin D2-d5, Leukotriene B4-d4, and Prostaglandin E1-d4 as internal standards (in 150 μL methanol) for recovery and quantitation and mixed thoroughly. The samples were then extracted for polyunsaturated fatty acid metabolites using C18 extraction columns as previously described^21,23–25,27,34^. Briefly, the internal standard spiked samples were applied to conditioned C18 cartridges, washed with 15% methanol in water followed by hexane, and then dried under vacuum. The cartridges were eluted with 2 volumes of 0.5 mL methanol with 0.1% formic acid. The eluate was dried under a gentle stream of nitrogen. The residue was redissolved in 50 μL methanol–25 mM aqueous ammonium acetate (1:1) and subjected to LC-MS/MS analysis.

HPLC was performed on a Prominence XR system (Shimadzu) using Luna C18 (3 μm, 2.1 × 150 mm) column. The mobile phase consisted of a gradient between A, methanol-water-acetonitrile (10:85:5 v/v), and B, methanol-water-acetonitrile (90:5:5 v/v), both containing 0.1% ammonium acetate. The gradient program with respect to the composition of B was as follows: 0–1 minute, 50%; 1–8 minutes, 50%–80%; 8–15 minutes, 80%–95%; and 15–17 minutes, 95%. The flow rate was 0.2 mL/min. The HPLC eluate was directly introduced to the electrospray ionization source of a QTRAP 5500 mass analyzer (Sciex) in the negative ion mode with following conditions: curtain gas: 35 psi, GS1: 35 psi, GS2: 65 psi, temperature: 600°C, ion spray voltage: −1500 V, collision gas: low, declustering potential: −60 V, and entrance potential: −7 V. The eluate was monitored by Multiple Reaction Monitoring (MRM) method to detect unique molecular ion–daughter ion combinations for each of the lipid mediators using a scheduled MRM around the expected retention time for each compound. Optimized collisional energies (18–35 eV) and collision cell exit potentials (7–10 V) were used for each MRM transition. Spectra of each peak detected in the scheduled MRM were recorded using enhanced product ion scan to confirm the structural identity. The data were collected using Analyst 1.7 software, and the MRM transition chromatograms were quantitated by MultiQuant software (both from Sciex). The internal standard signals in each chromatogram were used for normalization, recovery, as well as relative quantitation of each analyte.

LC-MS/MS data were analyzed using MetaboAnalyst 6.0^35^. Missing values were replaced with the estimated limit of detection (LoDs) (1/5 of the minimum positive value of each variable). Heatmaps were generated using the Euclidean distance measure and the Ward clustering algorithm following autoscaling of features without data transformation. Targeted parametric statistical analysis was also performed on a predetermined subset of metabolites of interest.

#### Muscle Force Testing:

These procedures are modified from Dellorusso et al. 2001. Mice were anesthetized with 2% Isoflurane to maintain a deep anesthesia throughout the experiment. Hindlimb fur was removed with clippers. The TA muscle was exposed by removing the overlying skin and outer fasciae. The distal TA tendon was isolated and the distal half of the TA was freed from adjacent muscles by carefully cutting fasciae without damaging muscle fibers. A 4–0 silk suture was tied around the distal TA tendon, and the tendon was severed from its bony insertion. The animal was then placed on a temperature-controlled platform warmed to maintain body temperature at 37°C. A 25-gauge needle was driven through the knee and immobilized to prevent the knee from moving. The tendon was tied securely to the lever arm of a servomotor via the suture ends (6650LR, Cambridge Technology). A continual drip of saline warmed to 37°C was administered to the TA muscle to maintain its temperature. The TA muscle was stimulated with 0.2 ms pulses via the peroneal nerve using platinum electrodes. Stimulation voltage and muscle length were adjusted for maximum isometric twitch force (P_t_). While held at optimal muscle length (L_o_), the muscle was stimulated at increasing frequencies until maximum isometric tetanic force (P_o_) was reached, typically at 200 Hz, with a one-minute rest period between each tetanic contraction. Muscle length was measured with calipers, based on well-defined anatomical landmarks near the knee and the ankle. Optimum fiber length (L_f_) was determined by multiplying Lo by the TA L_f_/L_o_ ratio of 0.6^36^. After the evaluation of isometric force, the TA muscle was removed from the mouse. The tendon and suture were trimmed from the muscle, and the muscle was weighed. After removal of TA muscles, deeply anesthetized mice were euthanized by the induction of a pneumothorax. Total muscle fiber cross-sectional area (CSA) of TA muscles was calculated by dividing muscle mass by the product of Lf and 1.06 mg/mm^3^, the density of mammalian skeletal muscle^37^. Specific Po was calculated by dividing P_o_ by muscle CSA.

### Statistics:

Data are presented as individual dot plots and the group means ± SEM. GraphPad Prism 10 was used for statistical analysis. Comparisons between two independent groups were performed using two-tailed unpaired t-tests, by one-way ANOVA followed by pairwise Holm-šidák post hoc tests for three or more groups, and by two-way ANOVA followed by pairwise Holm-šidák post hoc tests for experiments with ≥2 factors and ≥2 levels. In time-course experiments, multiple-comparisons testing was conducted using a single baseline control group. P≤0.05 were considered statistically significant.

## Results:

### Leukocyte-type 12/15-LOX is expressed in skeletal muscle tissue and *Alox15*^−/−^ mice are deficient in basal intramuscular lipid mediators:

**Figure 1:**
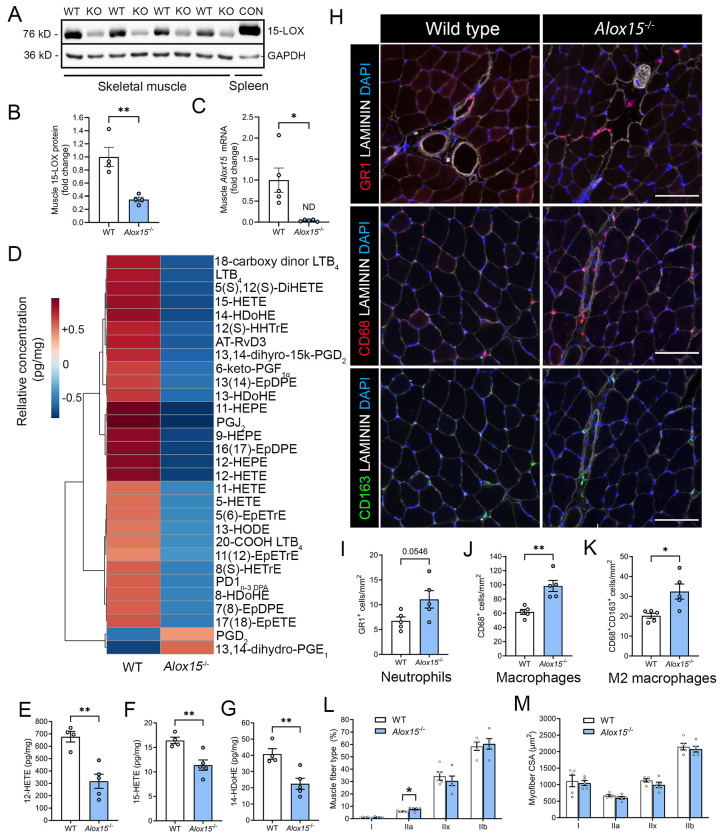
*Alox15*^−/−^ mice are deficient in intramuscular 12/15-LOX-derived lipid mediators and display chronic low-grade muscle inflammation. **A:** Western blot analysis of the abundance of the 12/15-LOX protein in TA muscle homogenates from WT and *Alox15*^−/−^ mice. A spleen homogenate from a WT mouse served as a positive control. **B:** Densitometry quantification of the abundance of the 12/15-LOX protein in the TA muscle. Protein expression was normalized to GAPDH abundance. **C:** *Alox15* mRNA expression in the TA muscle as determined by real-time quantitative reverse transcription PCR (RT-qPCR). Gene expression was normalized to *Gapdh*. **D:** Heatmap of the top 30 most differentially abundant lipid mediators TA muscle homogenates between WT and *Alox15*^−/−^ mice as identified by liquid chromatography-tandem mass spectrometry (LC-MS/MS). **E-G:** Quantification of the intramuscular concentration (pg/mg) of major 12/15-LOX metabolites including 12-hydroxy-eicosatetraenoic acid (12-HETE) (**E**), 15-hydroxy-eicosatetraenoic acid (15-HETE) (**F**), and 14-hydroxy-docosahexaenoic acid (14-HDoHE) (**G**) in TA muscle homogenates as determined by LC-MS/MS. **H:** TA muscle cross-sections were stained with primary antibodies against the neutrophil marker GR1, the pan monocyte/macrophage marker CD68, or the M2 macrophage marker CD163. Scale bars are 100 μm. **I-K:** Quantitative analysis of intramuscular numbers of neutrophils (GR1^+^ cells) (**I**), monocyte/macrophages (CD68^+^ cells) (**J**), and M2-like macrophages (CD68^+^CD163^+^ cells) (**K**). **L-M:** Quantification of percentage TA muscle fiber type (**L**) and fiber type specific myofiber cross-sectional area (CSA) (**M**) as determined by MuscleJ 1.0.2 software. Bars show the mean ± SEM of 4-5 mice per group (biological replicates) with dots representing data from each individual mouse. P-values were determined by two-tailed unpaired t-tests. *p<0.05, **p<0.01 for WT vs. *Alox15*^−/−^ mice.

Western blot analysis for the murine 12/15-LOX (15-LOX-1) protein detected a 76 kDa band in TA muscle homogenates from WT mice that matched that seen in WT spleen homogenates as a positive control (Fig. 1A). In contrast, *Alox15*^−/−^ mice had markedly reduced TA muscle 12/15-LOX protein expression (Fig. 1B). RT-qPCR also detected expression of *Alox15* mRNA in the TA muscle of WT mice, while its expression was undetectable in *Alox15*^−/−^ mice (Fig. 1C). LC-MS/MS analysis detected a total of 65 and 56 lipid mediators in TA muscle homogenates of WT and *Alox15*^−/−^ mice, respectively (Table S1). A heat map of the top 30 lipid mediators differing most between WT and *Alox15*^−/−^ mice is shown in Fig. 1D. Overall, 16 metabolites were significantly reduced (unadjusted p<0.05) in *Alox15*^−/−^ mice (Table S2). *Alox15*^−/−^ mice showed reduced intramuscular concentrations of 12/15-LOX metabolites of ARA (e.g., 12-HETE & 15-HETE), EPA (e.g., 12-HEPE), and DHA (e.g., 14-HDoHE) (Fig. 1E–G, Table S2). 15-HETE and 14-HDoHE are well-established intermediates in the biosynthesis of the lipoxin and maresin family of SPMs, respectively. However, downstream lipoxins (LXA_4_ and LXB_4_) and maresins [MaR1 and 7(S)-MaR1] were below the limits of detection of our LC-MS/MS assay. We did detect some other SPMs including RvD1, RvD6. AT-RvD3, and MaR1_n-3DPA_ (Table S1). But, of these only AT-RvD3 was significantly lacking in *Alox15*^−/−^ mice (Table S2). Interestingly, some prostaglandins with purported anti-inflammatory actions (e.g.,13,14dh-15k-PGD_2_ and PGJ_2_)^38^ were also reduced in concentrations in muscle of *Alox15*^−/−^ mice (Table S2). Overall, these data show that local 12/15-LOX activity is essential in maintaining a normal resting skeletal muscle lipid mediator profile.

### Basal skeletal muscle phenotype in WT and *Alox15*^−/−^ mice:

To examine the potential consequences of a lack of 12/15-LOX derived lipid mediators, muscle mass and strength were compared between WT and *Alox15*^−/−^ mice. *Alox15* knockout had no effect on body weight (Fig. S1A), absolute *in situ* TA muscle strength (Fig. S1B), or muscle strength when normalized to muscle size (maximal specific force) (Fig. S1C). Finally, although the mass of the quadriceps (QUAD) muscle was significantly reduced in *Alox15*^−/−^ mice, there was no such difference in mass of the other hind-limb muscles including the soleus (SOL), plantaris (PLA), tibialis anterior (TA), or gastrocnemius (GAST) (Fig. S1D). Overall, these data suggests that the development and maintenance of skeletal muscle tissue appears generally undisturbed in young adult whole-body 12/15-LOX knockout mice.

### *Alox15*^−/−^ mice show chronic low-grade skeletal muscle inflammation:

Some 12/15-LOX pathway metabolites (e.g., SPMs) have been implicated as playing an important counterregulatory role in the inflammatory response^20^. Therefore, we sought to quantify leukocyte numbers in TA muscles obtained from WT and *Alox15*^−/−^ mice (Fig. 1H). *Alox15*^−/−^ mice displayed a statistical trend (p=0.055) toward greater numbers of intramuscular PMNs (GR1^+^ cells) (Fig. 1I), as well as more total Mϕ (CD68^+^ cells) (Fig. 1J), and M2-like Mϕ (CD68^+^CD163^+^ cells) (Fig. 1K). Analysis of TA muscle fiber type profile revealed a higher proportion of type IIA myofibers in *Alox15*^−/−^ mice, but there was no difference in the percentage of type I, IIX, or IIB fibers. (Fig 1L). *Alox15*^−/−^ mice also did not differ significantly from WT mice in the mean cross-sectional area (CSA) of type I, IIA, IIX, or IIB myofibers (Fig. 1M). Representative fiber type staining is shown in Fig. S1E. Overall, these data show that a deficiency in *Alox15* results in chronic low-grade muscle inflammation and a shift in fiber type profile, but no overt evidence of myofiber atrophy.

### *Alox15*^−/−^ mice mount an exaggerated innate immune response to acute skeletal muscle injury:

**Figure 2:**
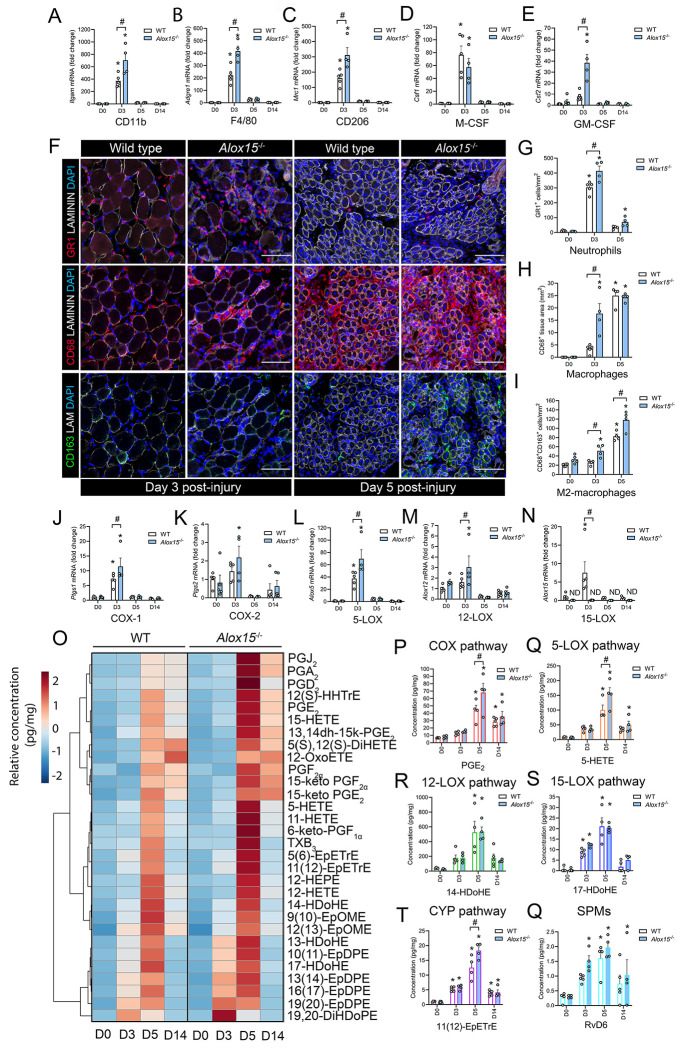
Leukocyte-type 12/15-LOX deficient mice display greater local inflammation and an imbalance of pro-inflammation vs. anti-inflammatory/pro-resolving lipid mediators following acute skeletal muscle injury. **A-E:** Tibialis anterior (TA) muscle mRNA expression of the pan myeloid cell marker CD11b (*Itgam*) (**A**), the pan monocyte/ Mϕ marker F4/80 (*Adgre1*) (**B**), the M2 Mϕ marker CD206 (*Mrc1*) (**C**), the hematopoietic growth factor M-CSF (*Csf1*) (**D**), and the hematopoietic growth factor GM-CSF (*Csf2*) (**E**) in wild type (WT) and *Alox15*^−/−^ mice at day 0 (D0), day 3 (D3), day 5 (D5), and day 14 (D14) following myofiber injury induced by intramuscular injection of 50 μL of 1.2% barium chloride (BaCl_2_). Expression of genes of interest was normalized to *Gapdh*. **F:** Immunofluorescence staining with primary antibodies against neutrophils (PMNs) (GR1), monocytes/Mϕ (CD68), and M2 Mϕ (CD163) in injured TA muscle of WT and *Alox15*^−/−^ mice on D3 and D5 post-injury. Cell nuclei were stained with DAPI and a primary antibody against laminin was used to identify muscle fiber boundaries. Scale bars are 100 μm. **G-I**: Quantification of PMNs (GR1^+^ cells/mm^2^) (**G**), monocytes/ Mϕ (CD68^+^ cells/mm^2^) (**H**), and M2 Mϕ (CD68^+^CD163^+^ cells/mm^2^) (**I**) at D0, D3, and D5 post-injury in WT and *Alox15*^−/−^ mice. **J-N:** TA mRNA expression of major lipid mediator biosynthesis enzymes including COX-1 (*Ptgs1*) (**J**), COX-2 (*Ptsg2*) (**K**), 5-LOX (*Alox5*) (**L**), 12-LOX (*Alox12*) (**M**), and 15-LOX (*Alox15*). **O:** Heatmap of the top 30 most differentially abundant lipid mediators between WT and *Alox15*^−/−^ mice as identified by liquid chromatography-tandem mass spectrometry (LC-MS/MS) analysis of TA muscle homogenates. **P-Q:** Quantification of major representative metabolites of the COX pathway (e.g., PGE_2_) (**P**), 5-LOX pathway (e.g., 5-HETE) (**Q**), 12-LOX pathway (e.g., 14-HDoHE) (**R**), 15-LOX pathway (e.g., 17-HDoHE) (**S**), CYP pathway [e.g., 11(12)-EpETrE) (**T**), and downstream bioactive SPMs (e.g., RvD6) (**Q**). Bars show the mean ± SEM of 4-5 mice per group (biological replicates) with dots representing data from each individual mouse. P-values were determined by two-way ANOVA followed by Holm-Šídák post-hoc tests. *p<0.05 vs. D0 and #p<0.05 for WT vs. *Alox15*^−/−^ mice.

To assess the impact of 12/15-LOX deficiency on the acute inflammatory response to myofiber injury, TA muscles were collected at day 3 (D3), day 5 (D5), and day 14 (D14) following intramuscular injection of barium chloride (BaCl_2_). Muscle mRNA expression of the pan myeloid cell marker CD11b (*Itgam*) (Fig 2A), the Mϕ marker F4/80 (*Adgre1*) (Fig 2B), and the M2-like Mϕ marker CD206 (*Mrc1*) (Fig. 2C) increased markedly (>100-fold) on D3 and were each relatively greater in *Alox15*^−/−^ vs. WT mice (Fig 2A–C). Local expressions of hematopoietic growth factors including macrophage colony-stimulating factors (M-CSF, *Csf1*) (Fig. 2D) and granulocyte-macrophage colony-stimulating factor (GM-CSF, *Csf2*) (Fig. 2E) also increased greatly on D3. Furthermore, GM-CSF, but not M-CSF, was also substantially greater in *Alox15*^−/−^ vs WT mice at this time-point (Fig 2E). 12/15-LOX knockout did not affect the induction of a range of other classical pro- and anti-inflammatory cytokines such as TNFα (*Tnf1*) (Fig S2A), IL-1β (*Il1b*) (Fig S2B), IL-6 (*Il6*) (Fig S2C), or IL-10 (*Il10*) (Fig S2D). Nevertheless, monocyte chemoattractant protein 1 (MCP-1, *Ccl2*) was significantly lower in *Alox15*^−/−^ vs. WT mice on D3 (Fig S2E). Interestingly, expression of insulin like growth factors (IGF-1, *Igf1*) (Fig. S2F), transforming growth factor beta (TGF-β, *Tgfb1*) (Fig. S2H), and arginase-1 (ARG-1, *Arg1*) (Fig. S2I) were each significantly greater in *Alox15*^−/−^ mice on D3. On the other hand, vascular endothelial growth factor (VEGF, *Vegfa*) was blunted in *Alox15*^−/−^ mice on D14 relative to WT controls (Fig S2J).

Immunofluorescent staining revealed that muscle infiltration of blood PMNs (GR1^+^ cells) peaked on D3 post-injury (Fig 2F). Additionally, *Alox15*^−/−^ mice showed relatively greater intramuscular numbers of PMNs at this time-point (Fig 2G). Intramuscular infiltration of Mϕ (CD68^+^ cells) peaked on D5 (Fig 2F). When compared to WT controls, *Alox15*^−/−^ mice showed an exaggerated CD68^+^ cell infiltration at D3, but similar intramuscular numbers of Mϕs by D5 (Fig. 2H). M2 Mϕ (CD68^+^CD163^+^ cells) numbers also peaked on D5 and were more numerous in *Alox15*^−/−^ mice at both D3 and D5 (Fig. 2I). These data show that *Alox15*^−/−^ mice exhibit an overall relatively greater acute inflammatory response when compared to WT mice.

#### Dysregulated local lipid mediator response to skeletal muscle injury in *Alox15*^−/−^ mice:

RT-qPCR analysis revealed that muscle mRNA expression of major lipid mediator biosynthetic enzymes including COX-1 (*Ptgs1*) (Fig. 2J), 5-LOX (*Alox5*) (Fig. 2L), 5-LOX activating protein (FLAP, *Alox5ap*) (**data not shown**), and 15-LOX (*Alox15*) (Fig 2N) all increased greatly on D3. As expected, *Alox15* mRNA expression was undetectable in *Alox15*^−/−^ mice at D3, D5, and D14 (Fig 2N). On the other hand, COX-1 (*Ptgs1*) (Fig. 2J), COX-2 (*Ptgs2*) (Fig. 2K), 5-LOX (*Alox5*) (Fig. 2L), FLAP (*Alox5ap*) (**data not shown**), and 12-LOX (*Alox12*) (Fig. 2M) were each more highly expressed in *Alox15*^−/−^ vs. WT mice on D3. Overall, these data show that *Alox15*^−/−^ mice appear to mount a compensatory transcriptional upregulation of other lipid mediator biosynthetic enzyme in the absence of leukocyte-type 12/15-LOX.

LC-MS/MS-based metabolomic profiling revealed that a total of 53, 72, and 48 lipid mediators increased (unadjusted p<0.05) in muscle tissue of WT mice at D3, D5, and D14 post-injury, respectively (Table S3). In contrast, a total of only 5, 0, and 2 analytes decreased in muscle tissue of WT mice at D3, D5, and D14, respectively (Table S3). A heatmap of the top 30 lipid mediators most influenced by BaCl_2_-induced muscle damage is shown in Fig. 2O. Following muscle injury there was increased local concentrations of many LC-PUFA metabolites of the COX pathway (e.g., PGE_2_), 5-LOX pathway (e.g., 5-HETE), 12-LOX pathway (e.g., 14-HDoHE), 15-LOX pathway (e.g., 17-HDoHE), and CYP pathway [e.g., 5(6-EpETrE)] (Fig. 2O). Some downstream SPMs including LXA_4_, RvD1, RvD2, and RvD6 were also detected at increased concentrations in regenerating muscle tissue on D5 and/or D14 (Table S3). When compared to WT controls, *Alox15*^−/−^ mice showed relatively greater concentrations of many n-6 ARA metabolites of the COX pathway (e.g., PGE_2_) (Fig. 2P & Fig S3A), the 5-LOX pathway (e.g., 5-HETE) (Fig. 2Q & Fig S3B), and the CYP pathway (e.g., [11(12)-EpETrE]) (Fig. 2T & Fig. S3E). In contrast, major n-3 DHA metabolites of 12-LOX pathway (e.g., 14-HDoHE) (Fig. 2R & Fig S3C) and 15-LOX pathway (e.g., 17-HDoHE) (Fig. 2S & Fig. S3D) were surprisingly detected at similar concentrations in WT and *Alox15*^−/−^ mice. Similarly, downstream n-3 series SPMs (e.g., RvD6) did not differ significantly between WT and *Alox15*^−/−^ mice (Fig. 2Q & Fig S3F). Surprisingly, concentrations of the n-6 ARA derived SPM LXA_4_ were significantly greater in *Alox15*^−/−^ vs. WT mice on D5 (Fig. S2F), as were levels of its biosynthetic intermediate 15-HETE (Fig. S2D). While E-series resolvins (e.g., RvE1) were not detected, the E-resolvin pathway marker 18-HEPE was also significantly greater in *Alox15*^−/−^ vs WT mice on D5 (Fig. S3F). Overall, these data reveal an absolute overabundance of pro-inflammatory n-6 metabolites of the COX and 5-LOX pathways, together with a relative deficiency of n-3 DHA derived anti-inflammatory/pro-resolving mediators of the 12/15-LOX pathways in *Alox15*^−/−^ mice. Nevertheless, some potential n-6 PUFA metabolites of the 15-LOX pathway, such as 15-HETE and LXA_4_ are greater in *Alox15*^−/−^ mice.

### Deleterious effects of *Alox15* deficiency on skeletal muscle regeneration:

**Figure 3:**
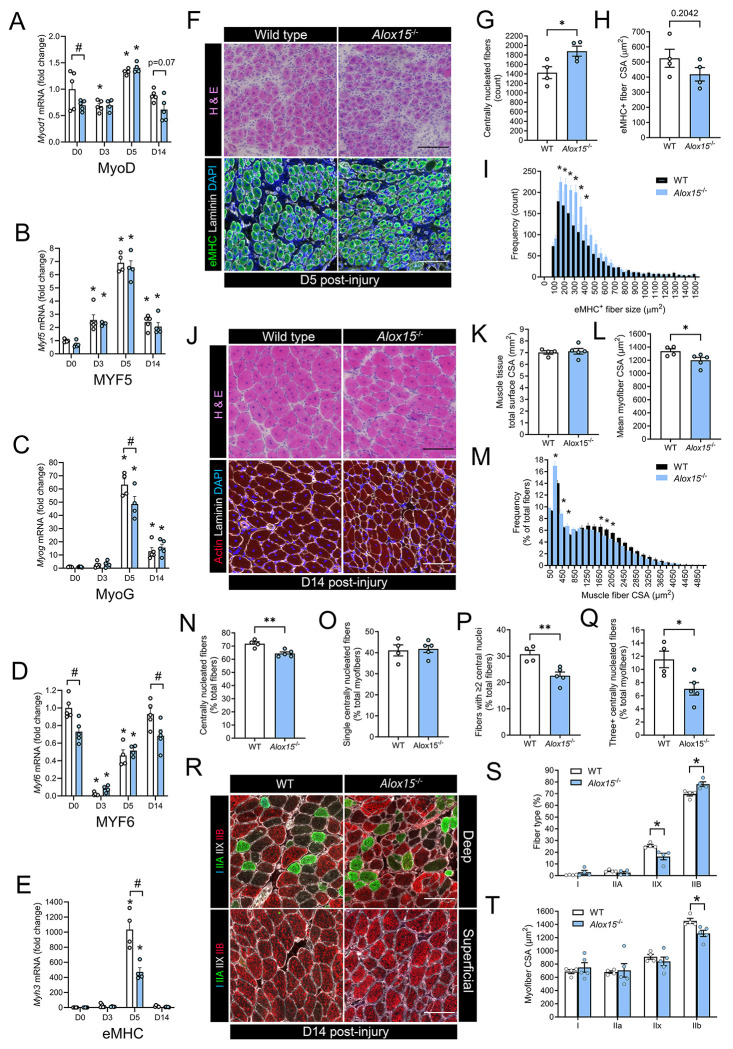
Deleterious effect of leukocyte-type 12/15-LOX deficiency on skeletal muscle regeneration: **A-E** Muscle mRNA expression of myogenic genes including MyoD (*Myod1*) (**A**), MYF5 (*Myf5*) (**B**), MyoG (*Myog*) (**C**), MYF6 (*Myf6*) (**D**), and eMHC (*Myh3*) **(E)** at day 3 (D3), day 5 (D5), and day 14 (D14) following muscle injury induced by intramuscular injection of BaCl_2_. Expression of genes of interest was normalized to *Rplp0*. **F:** Cross-sections of tibialis anterior (TA) muscles from wild type (WT) and *Alox15*^−/−^ mice obtained on D5 post-injury were stained with Hematoxylin & Eosin (H & E) or with primary antibodies against eMHC, laminin, and DAPI to assess regenerating muscle fiber morphology. Scale bars are 100 μm. **G-I:** Quantitative analysis of the absolute number of regenerating (centrally nucleated) myofibers (**G**), mean regenerating (eMHC^+^) myofiber cross-sectional area (CSA) (**H**), and frequency distribution of regenerating (eMHC+) myofiber CSA (**I**). **J:** TA muscle sections obtained on D14 post-injury were stained with H & E or with primary antibodies against actin, laminin, and DAPI for analysis of regenerating muscle fiber morphology. Scale bars are 100 μm. **K-M:** Quantification of the total TA muscle cross-sectional area (CSA) (**K**), mean TA muscle fiber CSA (**L**), and frequency distribution of myofiber CSA (**M**) within TA muscle cross-sections obtained from WT and *Alox15*^−/−^ mice on D14 post-injury. **N-Q:** Quantification of the percentage of centrally nucleated myofibers (**N**), and the proportion of regenerating muscle fibers containing one (**O**), two (**P**), or ≥three (**Q**) centrally located myonuclei in TA muscle cross-sections from WT and *Alox15*^−/−^ mice on D14 post-injury. **R:** TA muscle cross-sections obtained on day 14 post-injury were stained with primary antibodies against type I, IIA, and IIB myosin heavy chain (MyHC). Type IIX myofibers remain unstained (black). Scale bars are 100 μm. **S-T:** Quantification of percentage muscle fiber type profile (**S**) and fiber type specific myofiber CSA (**T**). Bars show the mean ± SEM of 4-5 mice per group (biological replicates) with dots representing data from each individual mouse. **A-E:** P-values were determined by two-way ANOVA followed by Holm-Šídák post-hoc tests. *p<0.05 vs. D0 and #p<0.05 for WT vs. *Alox15*^−/−^ mice. **F-T:** P-values were determined by two-tailed unpaired t-tests. *p<0.05, **p<0.01 for WT vs. *Alox15*^−/−^ mice.

To investigate whether 12/15-LOX deficiency might impact upon the efficiency of skeletal muscle regeneration we first measured mRNA expression of key myogenic genes throughout the time-course of muscle injury and regeneration. *Alox15*^−/−^ mice showed significantly lower basal muscle expression of myogenic differentiation 1 (MyoD, *Myod1*) (Fig. 3A) and myogenic regulatory factor 4 (MRF4, *Myf6*) (Fig. 3D), but similar expression of myogenic factor 5 (MYF5, *Myf5*) (Fig. 3B) and myogenin (MyoG, *Myog*) (Fig. 3C). Expression of *Myod1*, *Myf5*, and *Myog* each increased greatly on day 5 post-injury, while *Myf6* mRNA rather decreased far below baseline levels at this time-point. When compared to WT controls, expression of *Myog* was significantly blunted on day 5 post-injury in *Alox15*^−/−^ mice (Fig. 3C). On day 14 post-injury, muscle expression of *Myf6* was also lower in *Alox15*^−/−^ mice (Fig. 3D) and a similar statistical trend was also observed for *Myod1* (Fig. 3A). Muscle mRNA expression of the developmental embryonic myosin isoform (eMHC, *Myh3*) increased markedly on D5, and this response was also significantly diminished in *Alox15*^−/−^ mice (Fig. 3E). Overall, these data suggest overall suppressed local induction of myogenic genes in *Alox15*^−/−^ mice during the regenerative phase following acute muscle injury.

Histological analysis revealed that both WT and *Alox15*^−/−^ mice mounted a robust regenerative response on D5 as indicated by a predominance of many small myofibers with centrally located myonuclei that expressed eMHC (Fig. 3F). At this point, *Alox15*^−/−^ mice showed more numerous regenerating myofibers (Fig. 3G). Nevertheless, these fibers tended to be relatively smaller in size when compared to WT mice (Fig. 3H). Indeed, frequency distribution analysis showed many more eMHC^+^ myofibers with a CSA in the range of 150-400 μm^2^ in *Alox15*^−/−^ vs. WT mice (Fig 3I). Regenerating myofiber size increased markedly (>2-fold) in both WT and *Alox15*^−/−^ mice between D5 and D14 (Fig. 3J). On D14 TA muscles from *Alox15*^−/−^ mice were like WT in overall size (Fig. 3K). Nevertheless, the average myofiber CSA was significantly lower in *Alox15*^−/−^ mice (Fig. 3L). This was attributable to both a relatively greater proportion of very small myofibers and lower proportion of larger myofibers in *Alox15*^−/−^ mice (Fig. 3M). The overall proportion of centrally nucleated myofibers was also significantly reduced in *Alox15*^−/−^ mice at D14 (Fig 3N). Interestingly, there was no difference between groups in the proportion muscle fibers containing a single centrally located myonuclei (Fig. 3O). However, the proportion of more mature regenerating myofibers with two central nuclei (Fig. 3P), as well as those with ≥3 central nuclei (Fig. 3Q) were significantly reduced in *Alox15*^−/−^ mice. Muscle fiber type staining showed that, as expected, regenerating muscles of both WT and *Alox15*^−/−^ were predominantly comprised of fast twitch type II myofibers (IIB, IIX, and IIA) (Fig. 3R). When compared to WT mice, regenerating muscles from *Alox15*^−/−^ mice contained a relatively increased proportion of type IIB myofibers together with a corresponding reduction in the proportion of type IIX myofibers (Fig. 3S). Furthermore, the mean CSA of the type IIB myofiber population, which comprised most TA muscle fibers, was significantly reduced in *Alox15*^−/−^ mice (Fig. 3T). Overall, these data show that *Alox15*^−/−^ mice mount an impaired cellular and molecular regenerative response to muscle injury.

### *Alox15*^−/−^ Mϕ show heightened cytokine expression, defective M2 polarization, and an altered lipid mediator profile:

**Figure 4:**
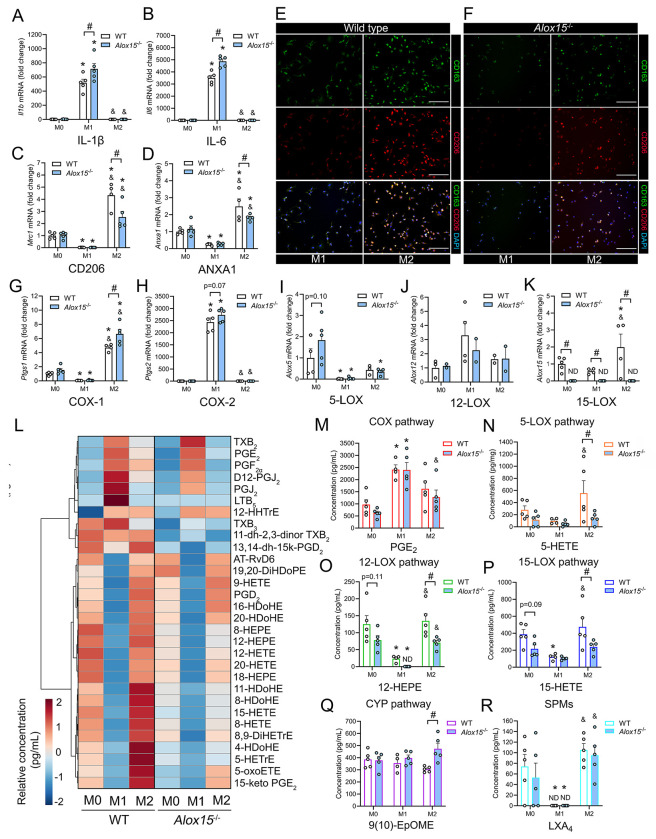
Bone marrow derived-macrophages obtained from *Alox15*^−/−^ mice display shifts in secreted lipid mediator profile and impaired M2 polarization. Bone-marrow-derived macrophages (Mϕ) (BMMs) obtained from wild type (WT) and 12/15-LOX deficient (*Alox15*^−/−^) mice were maintained in serum free media as naïve M0 Mϕ, or induced to differentiate into either M1 Mϕ by exposure to lipopolysaccharide (LPS) (100 ng/mL) and interferon gamma (IFN–*γ*) (20 ng/mL), or M2 Mϕ by exposure to interleukin-4 (IL-4) (20 ng/mL). Following 24 h of polarization mRNA expression levels of pro-inflammatory cytokines including interleukin-1β (*Il1b*) (**A**) and interleukin-6 (*Il6*) (**B**), or M2 Mϕ markers including CD206 (*Mrc1*) (**C**) and annexin A1 (*Anxa1*) (**D**) were measured by real-time quantitative reverse transcription PCR (RT-qPCR). **E-F:** BMMs from WT mice (**E**) and *Alox15*^−/−^ mice (**F**) were polarized to a M1 or M2 activation state for 48 h, fixed in 4% paraformaldehyde (PFA), and stained with primary antibodies against CD163 (green) and CD206 (red). Nuclei were counterstained by DAPI (blue). Scale bars are 200 μm. **G-K:** Gene expression of major lipid mediator biosynthesis enzymes including COX-1 (*Ptgs1*) (**G**), COX-2 (*Ptsg2*) (**H**), 5-LOX (*Alox5*) (**I**), platelet-type 12-LOX (*Alox12*) (**J**), and 15-LOX-1 (*Alox15*) (**K**) in M0, M1, and M2 Mϕ obtained from WT and *Alox15*^−/−^ mice following 24 h of polarization. **L:** A heatmap of the top 30 most differentially modulated lipid metabolites detected in conditioned cell culture media samples obtained from WT and *Alox15*^−/−^ M0, M1, and M2 Mϕ following 24 h incubation in serum free DMEM as assessed by targeted liquid chromatography-tandem mass spectrometry (LC-MS/MS). **M-R:** Concentrations of major representative lipid mediator metabolites of the cyclooxygenase (COX) pathway (e.g., PGE_2_) (**M**), 5-lipoxygenase (5-LOX) pathway (e.g., 5-HETE) (**N**), 12-lipoxygenase (12-LOX) pathway (e.g., 12-HEPE) (**O**), 15-lipoxygenase (15-LOX) pathway (e.g., 15-HETE) (**P**), cytochrome P450 (CYP 450) pathway [e.g., (9(10)-EpOME)] (**Q**), and downstream bioactive SPMs (e.g., LXA_4_) (**R**). Bars show the mean ± SEM of cells obtained from 5 mice per group (biological replicates) with dots representing BMMs from each individual mouse. P-values were determined by two-way ANOVA followed by Holm-Šídák post-hoc tests. *p<0.05 vs. M0, &p<0.05 vs. M1, and #p<0.05 for WT vs. *Alox15*^−/−^ cells.

We next investigated whether there may be inherent differences in the innate immune cell response that might contribute to poor muscle regeneration outcomes in *Alox15*^−/−^ mice. Naïve (M0) bone marrow-derived Mϕ (BMMs) were cultured *in vitro* and then polarized into pro-inflammatory M1 or anti-inflammatory M2 subsets by exposure to LPS + INF-γ or IL-4, respectively. RT-qPCR showed that expression of pro-inflammatory cytokines including IL-1β (*Il1b*) (Fig. 4A) and IL-6 (*Il6*) (Fig. 4B) were greatly increased following M1 polarization and that *Alox15*^−/−^ M1 Mϕ expressed even higher levels of these cytokines than WT cells (Fig. 4A & B). Other genes induced by M1 polarization, but not differing between WT and *Alox15*^−/−^ BMMs, included F4/80 (*Adgre1*), MCP-1 (*Ccl2*), TNFα (*Tnf*), M-CSF (*Csf1*), IL-10 (*Il10*) (Fig S4). Anti-inflammatory Mϕ markers including CD206 (*Mrc1*) (Fig. 4C) and annexin A1 (ANXA1) (Fig. 4D) were markedly upregulated following M2 polarization and these responses were blunted in *Alox15*^−/−^ cells. Expression of CD68 (*Cd68*) was also increased by M2 polarization, but did not differ between WT and *Alox15*^−/−^ BMMs (Fig. S4). Immunofluorescence staining also showed that *Alox15*^−/−^ Mϕ were less responsive to M2 polarization than WT Mϕ based on protein expression of anti-inflammatory Mϕ cell surface markers including CD163 and CD206 (Fig. 4E & F).

We next assessed the impact of polarization and 12/15-LOX knockout on lipid mediator biosynthesis pathways in BMMs. M1 polarization markedly increased Mϕ mRNA expression of *Ptgs2* (COX-2) (Fig. 4H), while reducing expression of *Ptgs1* (COX-1) (Fig. 4G), *Alox5* (5-LOX) (Fig. 4I), and Alox5ap (FLAP) (Fig. S4I). In contrast, M2 polarization increased Mϕ mRNA expression of *Ptgs1* (COX-1) (Fig. 4G), *Alox15* (15-LOX) (Fig. 4K), and Alox5ap (FLAP) (Fig. S4H), while reducing expression of *Ptgs2* (COX-2) (Fig. 4H) and *Alox5* (5-LOX) (Fig. 4I). Expression of *Alox12* (platelet-type 12-LOX) was not affected by Mϕ polarization (Fig. 4J). As expected, *Alox15* mRNA expression was undetected in *Alox15*^−/−^ M0, M1, and M2 Mϕ (Fig. 4K). *Alox15*^−/−^ BMMs expressed significantly higher levels of *Ptgs1* (COX-1) under M2 conditions (Fig. 4G), tended to express higher levels of *Ptgs2* (COX-2) under M1 conditions (Fig. 4H), and tended to express higher *Alox5* (5-LOX) under M0 conditions (Fig. 4I).

LC-MS/MS profiling of serum free conditioned cell culture media samples revealed that a total of 36 lipid mediators were altered following 24 h of polarization of WT Mϕ from a M1 to M2 phenotype (Table S5). A further 18, 17, and 26 lipid mediators differed between WT and *Alox15*^−/−^ Mϕ under identical M0, M1, and M2 conditions (Table S6). The top 30 extracellular lipid mediators influenced by polarization and/or *Alox15* deficiency are shown in Fig. 4L. Naïve M0 Mϕ from *Alox15*^−/−^ mice produced relatively lower concentrations than WT of some 12/15-LOX metabolites of n-6 ARA [e.g., 15-HETE, 15-Oxo-ETE, and 5(S),12(S)-DiHETE] and n-3 DHA (e.g., AT-RvD3 and RvD5) (Table S6). M1 polarization greatly increased extracellular concentrations of pro-inflammatory COX metabolites of n-6 ARA (e.g., PGE_2_) (Fig. 4M), Fig S4A). An exception was PGD_2_ which was markedly reduced by M1 polarization (Fig S4A). Prostaglandin biosynthesis was generally similar in WT and *Alox15*^−/−^ cells under M1 conditions (Fig. 4L). Nevertheless, *Alox15*^−/−^ M1 Mϕ did produce lower amounts of PGI_2_ (measured as 6-keto-PGF_1α_) than WT cells (Table S6). In WT Mϕ, M2 polarization decreased production of most COX metabolites (Fig S5A), while increasing production of many potential metabolites of the 5-LOX pathway (e.g., 5-HETE) (Fig. 4N, Fig S5B), the 12-LOX pathway (e.g., 12-HETE) (Fig. 4O, Fig S4C), and the 15-LOX pathway (e.g., 15-HETE) (Fig. 4P, Fig S5D). Production of many of these LOX metabolites was significantly diminished in *Alox15*^−/−^ Mϕ (Fig. 4N–P, Fig S5). M2 polarization of WT Mϕ also increased production of several di- and tri-hydroxy LOX metabolites (e.g., the SPMs) including LXA_4_, PD1, AT-PD1, AT-RvD6, and MaR1_n-3DPA_ (Fig 4R, Fig. S5F). However, of these only AT-PD1 and MaR1_n-3DPA_ were significantly lower concentrations in conditioned media samples obtained from *Alox15*^−/−^ Mϕ. While most lipid mediators were diminished by 12/15-LOX deficiency, *Alox15*^−/−^ M2 Mϕ produced greater amounts of some CYP pathway metabolites of linoleic acid (LA) [e.g., 9(10-EpOME)] (Fig. 4Q, Fig S5E). Overall, these data show that M1 polarization results in a COX dominated pro-inflammatory lipid mediator profile while M2 polarization leads to a shift to a LOX dominated anti-inflammatory/pro-resolving lipid signature. Furthermore, *Alox15*^−/−^ Mϕ produce lower amounts of many LOX-derived monohydroxy-PUFA metabolites under M2-polarizing conditions.

### Skeletal muscle cells secrete autocrine/paracrine 12/15-LOX metabolites that directly stimulate *in vitro* skeletal muscle cell growth and development.

**Figure 5:**
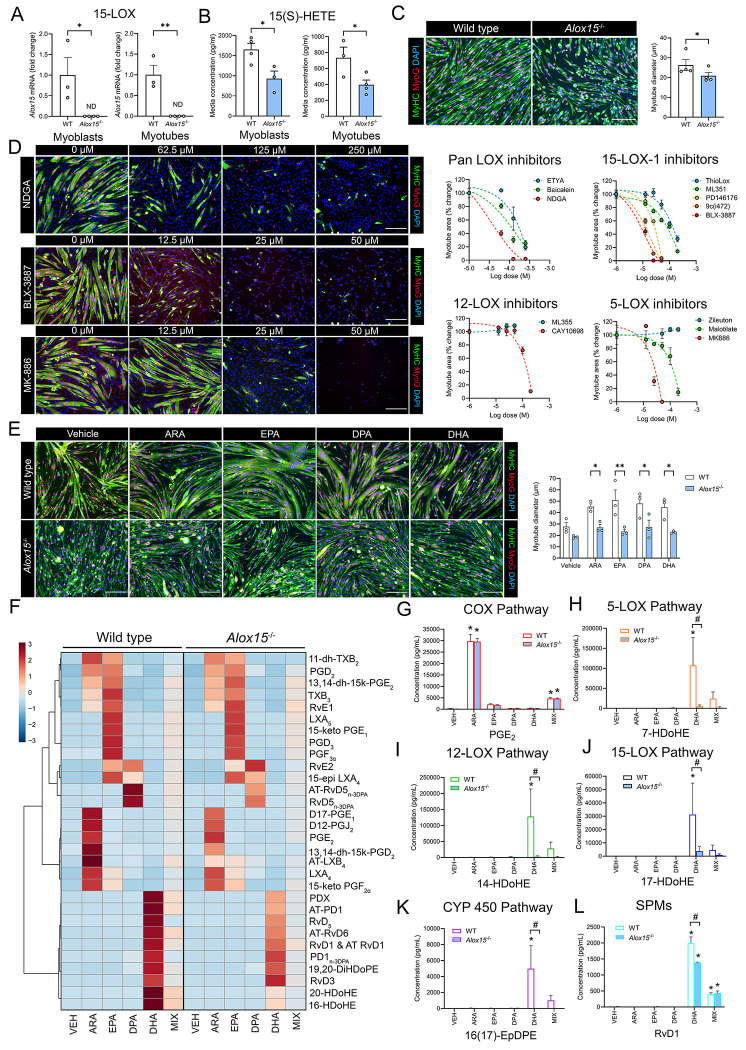
Leukocyte-type 12/15-LOX is a novel and direct determinant of myogenic progenitor cell fate. **A-B:** Primary myoblasts derived from wild type (WT) and *Alox15*^−/−^ mice were cultured *in vitro* and then induced to differentiate into myotubes. The expression level of *Alox15* mRNA was measured by RT-qPCR (**A**). The production of 15(S)-HETE in conditioned culture media obtained from primary myoblasts and myotubes was also quantified by ELISA (**B**). **C:** WT and *Alox15*^−/−^ myotubes were stained for myosin heavy chain (green) and myogenin (red) following 72 h of myogenic differentiation. Nuclei were counterstained by DAPI (blue). Mean myotube diameter was measured to assess the effect of *Alox15* deficiency on skeletal muscle cell growth and development. **D:** Murine C2C12 myoblasts were induced to differentiate in the presence of increasing doses of pharmacological LOX pathway inhibitors including pan LOX inhibitors (ETYA, baicalein, and NDGA), 15-LOX-1 specific inhibitors [BLX-3887, 9c(i472), and ThioLox], platelet type 12-LOX specific inhibitors (ML355 and CAY10698), and 5-LOX specific inhibitors (zileuton, malotilate, or MK886). Myotubes were stained for myosin heavy chain (green) and myogenin (red) following 72 h of myogenic differentiation. Nuclei were counterstained by DAPI (blue). Myotube formation was quantified as percentage of myosin^+^ cell area per field of view. **E:** Primary myoblasts from WT and *Alox15*^−/−^ mice were induced to differentiate for 3 days in the presence of a 25 μM dose of various individual long chain (LC) polyunsaturated fatty acids (PUFAs) including n-6 arachidonic acid (ARA), n-3 eicosapentaenoic acid (EPA), n-3 docosapentaenoic acid (DPA), and n-3 docosahexaenoic acid (DHA). Myotubes were stained for myosin heavy chain (green), myogenin (red), and DAPI (blue) for quantitative analysis of myotube diameter. **C-E:** Scale bars are 200 μm. **F-L:** Conditioned media was collected from myotube cultures receiving individual LC-PUFAs including ARA, EPA, DPA, and DHA at a dose of 25 μM or an equimolar mixture of 6.25 μM of each of these individual PUFAs reaching a total concentration of 25 μM. **F:** A heatmap of the top 30 most differentially regulated lipid mediators detected in conditioned culture media samples obtained from primary myotubes derived from WT and *Alox15*^−/−^ mice as measured by targeted liquid chromatography-tandem mass spectrometry (LC-MS/MS). **G-L:** Extracellular concentration major representative lipid mediator metabolites of the cyclooxygenase (COX) pathway (e.g., PGE_2_) (**G**), 5-lipoxygenase (5-LOX) pathway (e.g., 7-HDoHE) (**H**), 12-lipoxygenase (12-LOX) pathway (e.g., 14-HDoHE) (**I**), 15-lipoxygenase (15-LOX) pathway (e.g., 17-HDoHE) (**J**), cytochrome P450 (CYP 450) pathway [e.g., 16(17)-EpDPE)] (**K**), and downstream bioactive SPMs (e.g., RvD1) (**L**). Bars show the mean ± SEM of 4-5 mice per group (biological replicates) with dots representing data from each individual mouse. **A-E:** P-values were determined by two-tailed unpaired t-tests. *p<0.05 and *P<0.01 vs. WT myotubes. **G-L:** P-values were determined by two-way ANOVA followed by Holm-Šídák post-hoc tests. *p<0.05 vs. M0 and #p<0.05 for WT vs. *Alox15*^−/−^ cells.

We next examined whether muscle cells might also themselves express *Alox15* and whether a local deficiency of 12/15-LOX might directly influence muscle cell growth and development independent of immune-muscle cell crosstalk. Primary myoblasts were isolated from WT and *Alox15*^−/−^ mice and cultured *in vitro*. RT-qPCR detected the expression of *Alox15* mRNA in both WT myoblast and myotube cultures (Fig. 5A). In contrast, *Alox15* mRNA expression was undetectable in myoblast or myotube cultures obtained from *Alox15*^−/−^ mice (Fig. 5A). ELISA analysis of conditioned culture media samples revealed readily detectable extracellular concentrations of the 15(S)-HETE in conditioned culture media samples obtained from WT myoblasts and myotubes, but 15(S)-HETE was significantly reduced in conditioned media samples obtained from *Alox15*^−/−^ cells (Fig. 5B).

Immunocytochemistry analysis showed that following 72 h of myogenic differentiation that *Alox15*^−/−^ myoblasts fused to form smaller myotubes when compared to WT cells (Fig. 5C). Treatment of confluent C2C12 myoblasts at the onset of myogenic differentiation with the dual COX/LOX inhibitor eicosatetraynoic acid (ETYA), the pan LOX inhibitor nordihydroguaiaretic acid (NDGA), or the dual 12-LOX/15-LOX inhibitor baicalein each had marked direct suppressive effects on *in vitro* myotube formation (Fig. 5D, Fig S6A). Specific inhibitors of 15-LOX-1 including BLX-3887, 9c(i472), and PD146176 each also dramatically inhibited C2C12 myotube formation at relatively lower doses than pan LOX inhibitors (e.g., 10-20 μM) (Fig. 5D, Fig S6B). Two additional 15-LOX-1 specific inhibitors including ML351 and ThioLox (Fig S6B), as well as the platelet-type 12-LOX inhibitor CAY10698 (Fig S6D), also dramatically blocked myotube formation, but only at higher doses (100-200 μM) (Fig. 5D). Specific inhibitors of the 5-LOX pathway including zileuton and malotilate had little effect on myotube formation at low doses (10-20 μM) (Fig S6C). Nevertheless, malotilate (but not zileuton) did markedly blocked myotube formation at higher doses (100-200 μM) (Fig. 5D). Furthermore, MK-866, which interferes with 5-LOX activity indirectly via blockade of 5-LOX activating protein (FLAP) proved to be a highly potent inhibitor of C2C12 myotube formation at relatively low doses (e.g., 25-50 μM) (Fig. 5D, Fig S6C). Overall, these data suggest that leukocyte-type 12/15-LOX (15-LOX-1) and FLAP activity appear to be indispensable for successful muscle cell growth and development *in vitro*.

### LC-PUFA supplementation stimulates SPM biosynthesis and promotes muscle cell growth by a 12/15-LOX dependent pathway:

We next studied the potential effect of LC-PUFA supplementation on lipid mediator biosynthesis by isolated skeletal muscle cells. Primary myoblasts obtained from WT and *Alox15*^−/−^ mice were induced to differentiate for 72 h in the presence or absence of a 25 μM dose of pure individual LC-PUFAs including ARA, EPA, DPA, and DHA. Treatment with each of these LC-PUFAs alone greatly increased the mean diameter of WT myotubes (Fig. 5E). In contrast, primary myotubes from *Alox15*^−/−^ mice failed to respond to supplementation with any of these individual LC-PUFAs (Fig. 5E).

LC-MS/MS based profiling detected a total of 100 lipid mediator species present in conditioned culture media obtained from differentiating WT myoblasts in the absence of LC-PUFA supplementation (Table S1). Exogenous LC-PUFA treatment markedly increased extracellular concentrations of a wide range of lipid mediators in conditioned media samples (Fig. 5F). Supplementation with a 25 μM mixture of ARA, EPA, DPA, and DHA (6.25 μM each) showed the most diverse changes in culture media lipid mediator profile, altering the extracellular concentrations of a total of 40 different species of lipid metabolites (Table S7). Additionally, supplementation with a 25 μM dose of pure individual LC-PUFAs including ARA, EPA, DPA, and DHA altered the culture media concentrations of 28, 28, 25, and 32 lipid metabolites, respectively (Table S7).

ARA supplementation markedly increased concentrations of many metabolites of the COX pathway (e.g., PGE_2_) (Fig. 5G, Fig S7A). Of these, only PGF_2α_ differed between *Alox15*^−/−^ vs WT myotubes with lower levels produced by 12/15-LOX deficient cells (Fig S7A). Major 5-, 12-, and 15-LOX metabolites of ARA including 5-HETE (Fig. S7B), 12-HETE (Fig. S7C) and 15-HETE (Fig. S7D) were also greatly increased by ARA supplementation in WT myotubes. In contrast, 5-HETE, 12-HETE and 15-HETE did not increase in *Alox15*^−/−^ cells receiving ARA supplementation (Fig. S7C–D). The n-6 PUFA-derived SPM LXA_4_ was also increased following ARA treatment but was not influenced by *Alox15* deficiency (Fig. S7F). Nevertheless, the positional lipoxin isomer 15-epi-LXB_4_ was significantly lower in culture media samples obtained from *Alox15*^−/−^ myotubes (Table. S8). Supplementation with n-3 EPA greatly increased extracellular concentrations of series-3 COX metabolites (e.g., PGD_3_) (Table S7). EPA treatment also greatly increased culture media concentrations of E-series SPMs including 18-HEPE, RvE1, and RvE2 (Table S7). Overall, these responses were generally similar in WT and *Alox15*^−/−^ myotubes (Fig. S7F, Table S8). Supplementation with n-3 DPA increased concentrations of the di-hydroxy DPA products RvD5_n-3DPA_ and AT-RvD5_n-3DPA_ (Table S7), with comparable levels found in WT and *Alox15*^−/−^ muscle cells (Table S8). Supplementation with n-3 DHA greatly increased concentrations of monohydroxylated 5-, 12-, and 15-LOX metabolites of DHA including 7-HDoHE (Fig. 5H), 14-HDoHE (Fig. 5I), and 17-HDoHE (Fig. 5J). These responses were significantly diminished in *Alox15*^−/−^ muscle cells (Fig. 5H–J). DHA treatment also increased concentrations of some CYP450 metabolites (e.g., 16(17)-EpDPE) in WT, but not *Alox15*^−/−^ muscle cells (Fig. 5K). Downstream D-series SPMs including RvD1, RvD2, RvD3, RvD5, PDX, AT-PD1, AT-RvD6, and MaR2 were greatly increased in WT myotubes following DHA supplementation (Fig. 5L, Fig. S7F). In contrast, *Alox15*^−/−^ cultures showed lower extracellular concentrations than WT cells of RvD1, RvD2, RvD5, RvD6, PDX, and AT-PD1 (Fig. 5L, Fig S7F). Similar trends towards diminished levels of PD1, AT-RvD6, and MaR2 were also seen (Table S8). Overall, these data suggest that skeletal muscle cells directly produce LOX metabolites *in vitro*, some of which are dependent on the local expression of *Alox15*.

## Discussion:

In the current study, we investigated the role of leukocyte-type 12/15-LOX in skeletal muscle inflammation and regeneration in mouse and cell models. *Alox15* knockout dysregulated intramuscular lipid mediator profile leading to basal low-grade inflammation, exaggerated acute immune cell responses to tissue injury, and blunted skeletal muscle regeneration. Mechanistically, 12/15-LOX deficient Mϕ displayed impaired *in vitro* polarization to a pro-regenerative M2-like phenotype. Furthermore, 12/15-LOX deficient myogenic progenitor cells showed direct defects in *in vitro* myogenesis and were insensitive to the stimulatory effects of LC-PUFA supplementation upon muscle cell growth and differentiation. Overall, these data show that *Alox15* expression is essential for timely inflammation-resolution and regeneration following skeletal muscle injury via multiple mechanisms including both immunomodulation and direct determination of myogenic progenitor cell fate.

*Alox15*^−/−^ mice showed a dysregulated basal intramuscular lipid mediator profile that was associated with chronic low-grade skeletal muscle inflammation, as assessed by intramuscular Mϕ number. This finding is consistent with prior reports that *Alox15*^−/−^ mice show a chronic inflammatory state in other tissues, such as increased numbers of Mϕs in the skin^39^ and peritoneal cavity^40^. We hypothesize that basal muscle inflammation in *Alox15*^−/−^ mice could be attributed to a lack of 12/15-LOX derived lipid mediators with anti-inflammatory actions. In this regard, our LC-MS/MS data are generally consistent with a prior report that *Alox15*^−/−^ mice show a basal deficiency of some anti-inflammatory 12/15-LOX metabolites in the skin^39^. Overall, these data suggest that a lack of anti-inflammatory LC-PUFA metabolites may drive chronic low-grade skeletal muscle inflammation in young adult *Alox15*^−/−^ mice similar to our previous observation in aging WT mice^23^.

Chronic low-grade inflammation of skeletal muscle has been implicated in muscle atrophy^41–43^. However, we found little evidence indicative of muscle wasting or weakness in young adult male *Alox15*^−/−^ mice. Nevertheless, we did observe a significant increase in fast-oxidative-glycolytic (type IIA) myofibers in the TA muscle of *Alox15*^−/−^ mice. The potential role of 12/15-LOX activity in muscle fiber type determination remains elusive. When compared to the predominantly fast-twitch TA and EDL muscles, the predominantly slow twitch soleus muscle has been reported to be relatively enriched in 12/15-LOX metabolites^44–46^. Consistently, treatment with RvD2 was found to directly induce satellite cells to differentiate into slow-oxidative (type I) myotubes *in vitro*^44^. In contrast, daily IP injection of RvD2 increased fast-glycolytic fibers (type IIB) fibers in the regenerating TA muscle^44^. The precise role 12/15-LOX in fiber type determination may be muscle-specific, hence, the impact of 12/15-LOX deficiency on the fiber type of different muscles such as the slow-oxidative soleus warrants further study.

We found that *Alox15*^−/−^ mice showed an exaggerated innate immune response to skeletal muscle injury. These data are consistent with prior studies showing that *Alox15*^−/−^ mice mount greater inflammatory responses in several other models (e.g., ^40,47–52^). In contrast, some other experimental models have shown, paradoxically, that *Alox15*^−/−^ mice rather show blunted inflammation (e.g., ^53–59^). 12/15-LOX deficiency differentially impacted a range of inflammatory cytokine/chemokines in the current study. While many classical examples (e.g., TNFα, IL-1β, Il-6, and IL-10) were unaffected, *Alox15*^−/−^ mice showed a far greater local increase in GM-CSF expression following muscle injury. This is consistent with a prior study showing that GM-CSF was one of the most upregulated cytokines in peritoneal Mϕ isolated from *Alox15*^−/−^ mice^40^. On the other hand, we found the MCP-1 was less robustly induced in *Alox15*^−/−^ mice following muscle injury. This finding is consistent with prior studies showing that 12/15-LOX is an important signal driving Mϕ expression of MCP-1^60,61^. Notably, MCP-1 plays a crucial role in recruiting blood monocytes to injured skeletal muscle^62,63^. A blunted MCP-1 response in *Alox15*^−/−^ mice has been previously suggested as a potential mechanism to explain reduced monocyte recruitment^61^. Therefore, the increased early Mϕ infiltration of injured muscle in *Alox15*^−/−^ mice observed in the current study is likely driven by distinct mechanisms independent of MCP-1.

Administration of pharmacological doses of D-series SPMs such as RvD1^23,24^, AT-RvD1^26^, RvD2^22,44,64^, and MaR1^21^ has each recently been shown to limit inflammation, expedite its timely resolution, and stimulate myofiber regeneration following acute skeletal muscle injury. Long term systemic injection of the D-series SPM RvD2 also improved muscle regeneration in the *mdx* mouse model of muscular dystrophy^65,66^. Daily oral gavage with the D-series SPM protectin DX (PDX) was recently reported to be able to protect against development of age-associated musculoskeletal frailty^67^. While most work so far has focused on the n-3 DHA derived D-series SPMs, the E-series resolvin E1 (RvE1) has also been reported to protect muscle cells *in vitro* against inflammation and atrophy induced by lipopolysaccharide (LPS)^68^. Based on these prior studies we hypothesized that the heightened acute immune response following muscle injury in *Alox15*^−/−^ mice might be associated with a lack of SPMs and/or their pathway markers. Consistent with our prior studies^23,24^, we observed clear increases in local concentrations of 14-HDoHE and 17-HDoHE following muscle injury in WT mice. We also detected increased concentrations of some downstream DHA-derived SPMs including RvD1, RvD6, AT-RvD3, and MaR1. Surprisingly, absolute intramuscular concentrations of these analytes were clearly not lacking in *Alox15*^−/−^ mice. Nevertheless, the ratio of DHA derived SPMs and their pathway markers (e.g., 14-HDoHE and 17-HDoHE) relative to pro-inflammatory eicosanoids (e.g., PGE_2_) was reduced in 12/15-LOX deficient mice. As such, a relative deficiency of pro-resolving lipid mediators in combination with an absolute overabundance of pro-inflammatory eicosanoids may still contribute to the observed exaggerated acute immune cell responses in the current study.

Most of the studies conducted so far have focused on the impact of D-series SPMs on skeletal muscle injury and repair. Nevertheless, intravenous injection of the n-6 ARA-derived SPM LXA_4_ was also found to limit skeletal muscle inflammation following ischemia reperfusion injury in rats^69^. Consistent with our prior studies^23,24^, both LXA_4_ and its precursor molecule 15-HETE were greatly elevated after muscle injury in the current study. Surprisingly, 15-HETE and LXA_4_ were both present at greater concentrations in *Alox15*^−/−^ mice when compared to WT controls. This might be explained, in part, by the existence of alternative 15-LOX-1 independent routes of lipoxin biosynthesis. For example, cells expressing 5-LOX can convert ARA to leukotriene A_4_ (LTA_4_) which can be taken up by platelet-type 12-LOX expressing cells and converted to LXA_4_ and LXB_4_^70^. We found that both 5-LOX (*Alox5*) and platelet-type 12-LOX (*Alox12*) were more highly expressed in *Alox15*^−/−^ mice following muscle injury. Thus, the 5-LOX/12-LOX pathway may contribute to lipoxin biosynthesis in injured muscle. Prior studies have further shown that, substantial quantities of 15-HETE can be formed via COX-1 activity in 12/15-LOX knockout Mϕs, especially when 5-LOX was inactivated in parallel^71^. Indeed, herein COX-1 was more highly expressed in *Alox15*^−/−^ mice following muscle injury. Therefore, the COX-1 pathway might be an additional important contributor to 15-HETE and/or LXA_4_ biosynthesis in injured skeletal muscle.

We observed that BMMs obtained from *Alox15*^−/−^ mice exhibited exaggerated M1 polarization as well as impaired M2 polarization, and this was associated with underlying defects in lipid mediator class switching. These findings are consistent with prior studies reporting that certain 12/15-LOX metabolites, such as RvD1, can induce M2 Mϕ polarization^72^. Additionally, these findings agree with prior reports that M2 Mϕs may produce relatively greater concentrations of 12/15-LOX pathway metabolites when compared to M1 Mϕ^73,74^. While it was originally suggested that peritoneal Mϕ obtained from *Alox15*^−/−^ mice were indistinguishable from WT^30^, subsequent research showed that *Alox15*^−/−^ peritoneal Mϕ are greatly impaired in their ability to phagocytose apoptotic cells (efferocytosis)^48,75,76^. Moreover, consistent with our data, 12/15-LOX deficient peritoneal Mϕ were previously reported to be deficient in induction of the scavenger receptor CD36 in response to treatment with the anti-inflammatory cytokine IL-4^77^. Overall, these data suggest that impaired Mϕ functions may contribute, in part, to delayed inflammation-resolution in 12/15-LOX knockout mice.

We also found that *Alox15*^−/−^ mice displayed impaired *in vivo* myofiber regeneration. This finding is consistent with prior reports that *Alox15*^−/−^ mice display poor epithelial wound healing^78,79^. In contrast, *Alox15*^−/−^ mice have rather been found to be protected against inflammation-induced cardiac muscle damage^53–56,80^. The apparent overall deleterious effects of 12/15-LOX deficiency on skeletal muscle regeneration in the current study may be related to dysregulated immune-muscle cell interactions. PMNs can inflict secondary myofiber injury and delay myofiber regeneration following muscle injury^81^. Therefore, an excessive intramuscular PMN presence and impaired clearance from the site of injury in *Alox15*^−/−^ mice may have exacerbated local tissue damage and impeded myofiber regeneration. Blood monocytes are known to play an important supportive role in skeletal muscle regeneration, in part, by engulfing and clearing necrotic myofiber debris^82^. Nevertheless, earlier than normal peak blood monocyte/M1 Mϕ infiltration in *Alox15*^−/−^ mice may have negatively impact tissue repair, especially if their phagocytic capacity is impaired^48,75,76^. Surprisingly given that *Alox15*^−/−^ BMMs display defective M2 polarization *in vitro*, we observed greater numbers of M2-like CD163^+^ Mϕ within regenerating muscles of *Alox15*^−/−^ mice. M2 macrophages have generally been thought to play a beneficial role in skeletal muscle regeneration^83–85^. Nevertheless, it has also been reported that *Cd163*^−/−^ mice display improved skeletal muscle regeneration in a model of limb ischemia^86^. Another recent study suggested that depletion of CD206^+^ M2-like Mϕ also enhanced muscle regeneration following cardiotoxin induced injury^87^. Therefore, it is possible that an earlier than normal local transition of the greater numbers of infiltrating blood monocytes to M2 Mϕ in *Alox15*^−/−^ mice could interfere with muscle regeneration via multiple mechanisms^86,87^.

In addition to evidence of dysregulated immune-muscle cell crosstalk in 12/15-LOX deficient mice, we found that myogenic progenitor cells isolated from muscle tissue of *Alox15*^−/−^ mice showed marked impairments in their ability to form mature myotubes when cultured *in vitro*. Pharmacological LOX inhibitors also proved to be robust inhibitors of myotube formation in WT muscle cells. Amongst the pan LOX inhibitors tested, NDGA was the most potent inhibitor of myogenesis in our hands. This finding is consistent with a previously published paper showing that NDGA treatment resulted in severe dose-dependent inhibition of C2C12 myoblast differentiation^88^. Interestingly, the authors concluded that this marked inhibitory effect on *in vitro* myogenesis was independent of LOX since selective inhibitors of 5-LOX and platelet-type 12-LOX did not mimic the suppressive effects of NDGA^88^. Nevertheless, selective inhibitors of 15-LOX-1 were not tested^88^. When combined with our results, it appears likely that the deleterious effects of NDGA on myotube formation is primarily attributable to inhibition of 15-LOX-1. We found that the FLAP inhibitor MK886 also blocked C2C12 myotube formation, suggesting that FLAP activity is also indispensable for myotube formation. Interestingly, leukocyte FLAP activity has been previously shown to be required for the sequential 5-LOX mediated oxygenation of 15-HETE and/or 17-HDoHE to form downstream di- and tri-hydroxylated SPMs^89–91^.

It has been previously suggested that 15-HETE, the primary 15-LOX metabolite of n-6 ARA, is a cachexic factor produced by skeletal muscle cells that promotes protein degradation^92–95^. Nevertheless, consistent with prior published work by us and others^33,96–100^, ARA supplementation greatly stimulated *in vitro* myogenesis of WT myoblasts in the current study. In contrast, *Alox15*^−/−^ myoblasts were unresponsive to the stimulatory effects of ARA on myotube formation, and this was associated with lower 15-HETE levels. Consistent with some prior studies, we found that supplementation with n-3 LC-PUFAs including EPA, DPA, and DHA each also markedly stimulated *in vitro* myotube formation^97,101–106^. Like ARA, the stimulatory actions of n-3 LC-PUFAs on *in vitro* myogenesis were also dependent on 12/15-LOX activity. A lack of muscle cell growth in *Alox15*^−/−^ myoblasts in response to n-3 DHA supplementation was associated with diminished extracellular concentrations of many 12/15-LOX metabolites of DHA including several D-series SPMs. Overall, these data are consistent with recent reports by us and others that RvD1^24^, RvD2^44,65,66^, RvD3^107^, MaR1^21^, and RvE1^68^ have direct pro-myogenic and/or anti-catabolic effects on skeletal muscle cells cultured *in vitro*.

Taken together, our data strongly demonstrate that leukocyte-type 12/15-LOX is an important determinant of timely resolution of inflammation and myofiber regeneration following skeletal muscle injury. Consistent with previously published work, our data suggest that *Alox15* is important for Mϕ polarization to pro-resolutive and tissue reparative phenotype. Finally, we report a novel and direct role of *Alox15* as a previously unknown important intrinsic determinant of myogenic progenitor cell fate.

## Supplementary Material

Supplement 1

Supplement 2

Supplement 3

Supplement 4

Supplement 5

Supplement 6

Supplement 7

Supplement 8

9

## Figures and Tables

**Table 1.** Primer sequences used for RT-qPCR.

Gene	Primer	Sequence
*Gapdh*	F	CCCTTAAGAGGGATGCTGCC
	R	CAGGGATGATGTTCTGGGCA

*Tbp*	F	CCTTGTACCCTTCACCAATGAC
	R	ACAGCCAAGATTCACGGTAGA

*Rplp0*	F	GGCCCTGCACTCTCGCTTTC
	R	TGCCAGGACGCGCTTGT

*Itgam*	F	TGGCCTATACAAGCTTGGCTTT
	R	AAAGGCCGTTACTGAGGTGG

*Adgre1*	F	CCAGGAGTGGAATGTCAAGATGT
	R	GCAGACTGAGTTAGGACCACA

*Cd68*	F	ACTGGTGTAGCCTAGCTGGT
	R	CCTTGGGCTATAAGCGGTCC

*Mrc1*	F	GGCTGATTACGAGCAGTGGA
	R	CATCACTCCAGGTGAACCCC

*Cd163*	F	TCTCCTGGTTGTAAAAGGTTTGT
	R	CAGTTGTTTTCACCACCCGC

*Ptgs1*	F	CCAGAGTCATGAGTCGAAGGA
	R	CCTGGTTCTGGCACGGATAG

*Ptgs2*	F	AGCCAGGCAGCAAATCCTT
	R	CAGTCCGGGTACAGTCACAC

*Alox5*	F	ATTGCCATCCAGCTCAACCA
	R	ACTGGAACGCACCCAGATTT

*Alox5ap*	F	GCATGAAAGCAAGGCGCATA
	R	GCAGGGATTGGCAGTGTAGA

*Alox12*	F	GGATCCCTCAACCTAGTGCG
	R	AGTCAAACTCCTCCTCCTTGC

*Alox15*	F	TGATGACTTGGCTGAGCGAG
	R	TTCCCACCACGTACCGATTC

*Csf1*	F	GACCAGAGGACAGCTCCCTGA
	R	GGAGAGGGTAGTGGTGGATGT

*Csf2*	F	ACATGCCTGTCACGTTGAATG
	R	AAATTGCCCCGTAGACCCTG

*Tnf*	F	ATGGCCTCCCTCTCATCAGT
	R	TGGTTTGCTACGACGTGGG

*Il6*	F	TCCGGAGAGGAGACTTCACA
	R	TTGCCATTGCACAACTCTTTTCT

*Il1b*	F	GCCACCTTTTGACAGTGATGAG
	R	GACAGCCCAGGTCAAAGGTT

*Ccl2*	F	AGCTGTAGTTTTTGTCACCAAGC
	R	GACCTTAGGGCAGATGCAGT

*Il10*	F	GGCGCTGTCATCGATTTCTC
	R	ATGGCCTTGTAGACACCTTGG

*Igf1*	F	TGTACTGTGCCCCACTGAAG
	R	ATAGGGACGGGGACTTCTGA

*Egf*	F	GGACAGACAGTGGGAAGTCTG
	R	ATCCCTACGTCCGTCCAGAA

*Tgfb1*	F	ACTGGAGTTGTACGGCAGTG
	R	GGGGCTGATCCCGTTGATT

*Arg1*	F	AGAGATTATCGGAGCGCCTT
	R	GGTCTCTCACGTCATACTCTGTTTC

*Vegfa*	F	CTCCACCATGCCAAGTGGTC
	R	GTCCACCAGGGTCTCAATCG

## References

[1] Orimo S, Hiyamuta E, Arahata K, Sugita H. Analysis of inflammatory cells and complement C3 in bupivacaine-induced myonecrosis. Muscle Nerve. 1991;14(6):515–20. 1852158 10.1002/mus.880140605

[2] Fielding RA, Manfredi TJ, Ding W, Fiatarone MA, Evans WJ, Cannon JG. Acute phase response in exercise. III. Neutrophil and IL-1 beta accumulation in skeletal muscle. Am J Physiol. 1993;265(1 Pt 2):R166–72. 8342683 10.1152/ajpregu.1993.265.1.R166

[3] Smith JK, Grisham MB, Granger DN, Korthuis RJ. Free radical defense mechanisms and neutrophil infiltration in postischemic skeletal muscle. Am J Physiol. 1989;256(3 Pt 2):H789–93. 2923239 10.1152/ajpheart.1989.256.3.H789

[4] Pizza FX, Koh TJ, McGregor SJ, Brooks SV. Muscle inflammatory cells after passive stretches, isometric contractions, and lengthening contractions. J Appl Physiol (1985). 2002;92(5):1873–8. 11960936 10.1152/japplphysiol.01055.2001

[5] Pizza FX, Hernandez IJ, Tidball JG. Nitric oxide synthase inhibition reduces muscle inflammation and necrosis in modified muscle use. J Leukoc Biol. 1998;64(4):427–33. 9766622

[6] Robertson TA, Maley MA, Grounds MD, Papadimitriou JM. The role of macrophages in skeletal muscle regeneration with particular reference to chemotaxis. Exp Cell Res. 1993;207(2):321–31. 8344384 10.1006/excr.1993.1199

[7] Dyall SC, Balas L, Bazan NG, Brenna JT, Chiang N, da Costa Souza F, Polyunsaturated fatty acids and fatty acid-derived lipid mediators: Recent advances in the understanding of their biosynthesis, structures, and functions. Prog Lipid Res. 2022;86:101165. 35508275 10.1016/j.plipres.2022.101165 PMC9346631

[8] Kuhn H, Banthiya S, van Leyen K. Mammalian lipoxygenases and their biological relevance. Biochimica Et Biophysica Acta. 2015;1851(4):308–30. 25316652 10.1016/j.bbalip.2014.10.002 PMC4370320

[9] Singh NK, Rao GN. Emerging role of 12/15-Lipoxygenase (ALOX15) in human pathologies. Prog Lipid Res. 2019;73:28–45. 30472260 10.1016/j.plipres.2018.11.001 PMC6338518

[10] Kuhn H, Banthiya S, van Leyen K. Mammalian lipoxygenases and their biological relevance. Biochim Biophys Acta. 2015;1851(4):308–30. 25316652 10.1016/j.bbalip.2014.10.002 PMC4370320

[11] Powell WS, Rokach J. Biosynthesis, biological effects, and receptors of hydroxyeicosatetraenoic acids (HETEs) and oxoeicosatetraenoic acids (oxo-ETEs) derived from arachidonic acid. Biochim Biophys Acta. 2015;1851(4):340–55. 25449650 10.1016/j.bbalip.2014.10.008 PMC5710736

[12] Panigrahy D, Gilligan MM, Serhan CN, Kashfi K. Resolution of inflammation: An organizing principle in biology and medicine. Pharmacol Ther. 2021;227:107879. 33915177 10.1016/j.pharmthera.2021.107879

[13] Samuelsson B, Dahlen SE, Lindgren JA, Rouzer CA, Serhan CN. Leukotrienes and lipoxins: structures, biosynthesis, and biological effects. Science. 1987;237(4819):1171–6. 2820055 10.1126/science.2820055

[14] Fierro IM, Colgan SP, Bernasconi G, Petasis NA, Clish CB, Arita M, Lipoxin A4 and aspirin-triggered 15-epi-lipoxin A4 inhibit human neutrophil migration: comparisons between synthetic 15 epimers in chemotaxis and transmigration with microvessel endothelial cells and epithelial cells. J Immunol. 2003;170(5):2688–94. 12594298 10.4049/jimmunol.170.5.2688

[15] Maddox JF, Serhan CN. Lipoxin A4 and B4 are potent stimuli for human monocyte migration and adhesion: selective inactivation by dehydrogenation and reduction. J Exp Med. 1996;183(1):137–46. 8551217 10.1084/jem.183.1.137 PMC2192402

[16] Godson C, Mitchell S, Harvey K, Petasis NA, Hogg N, Brady HR. Cutting edge: lipoxins rapidly stimulate nonphlogistic phagocytosis of apoptotic neutrophils by monocyte-derived macrophages. J Immunol. 2000;164(4):1663–7. 10657608 10.4049/jimmunol.164.4.1663

[17] Hong S, Gronert K, Devchand PR, Moussignac RL, Serhan CN. Novel docosatrienes and 17S-resolvin generated from docosahexaenoic acid in murine brain, human blood, and glial cells. Autacoids in anti-inflammation. J Biol Chem. 2003;278(17):14677–87. 12590139 10.1074/jbc.M300218200

[18] Sun YP, Oh SF, Uddin J, Yang R, Gotlinger K, Campbell E, Resolvin D1 and its aspirin-triggered 17R epimer. Stereochemical assignments, anti-inflammatory properties, and enzymatic inactivation. J Biol Chem. 2007;282(13):9323–34. 17244615 10.1074/jbc.M609212200

[19] Serhan CN, Yang R, Martinod K, Kasuga K, Pillai PS, Porter TF, Maresins: novel macrophage mediators with potent antiinflammatory and proresolving actions. J Exp Med. 2009;206(1):15–23. 19103881 10.1084/jem.20081880 PMC2626672

[20] Serhan CN. Pro-resolving lipid mediators are leads for resolution physiology. Nature. 2014;510(7503):92–101. 24899309 10.1038/nature13479 PMC4263681

[21] Castor-Macias JA, Larouche JA, Wallace EC, Spence BD, Eames A, Duran P, Maresin 1 repletio improves muscle regeneration after volumetric muscle loss. Elife. 2023;12. 10.7554/eLife.86437 PMC10807862 38131691

[22] Giannakis N, Sansbury BE, Patsalos A, Hays TT, Riley CO, Han X, Dynamic changes to lipid mediators support transitions among macrophage subtypes during muscle regeneration. Nat Immunol. 2019;20(5):626–36. 30936495 10.1038/s41590-019-0356-7 PMC6537107

[23] Markworth JF, Brown LA, Lim E, Castor-Macias JA, Larouche J, Macpherson PCD, Metabolipidomic profiling reveals an age-related deficiency of skeletal muscle pro-resolving mediators that contributes to maladaptive tissue remodeling. Aging Cell. 2021;20(6):e13393. 34075679 10.1111/acel.13393 PMC8208786

[24] Markworth JF, Brown LA, Lim E, Floyd C, Larouche J, Castor-Macias JA, Resolvin D1 supports skeletal myofiber regeneration via actions on myeloid and muscle stem cells. JCI Insight. 2020;5(18). 10.1172/jci.insight.137713 PMC7526543 32750044

[25] Vella L, Markworth JF, Farnfield MM, Maddipati KR, Russell AP, Cameron-Smith D. Intramuscular inflammatory and resolving lipid profile responses to an acute bout of resistance exercise in men. Physiol Rep. 2019;7(13):e14108. 31257737 10.14814/phy2.14108 PMC6599756

[26] Turner TC, Pittman FS, Zhang H, Hymel LA, Zheng T, Behara M, Improving Functional Muscle Regeneration in Volumetric Muscle Loss Injuries by Shifting the Balance of Inflammatory and Pro-Resolving Lipid Mediators. bioRxiv. 2024.

[27] Markworth JF, Vella L, Lingard BS, Tull DL, Rupasinghe TW, Sinclair AJ, Human inflammatory and resolving lipid mediator responses to resistance exercise and ibuprofen treatment. Am J Physiol Regul Integr Comp Physiol. 2013;305(11):R1281–96. 24089379 10.1152/ajpregu.00128.2013 PMC3882565

[28] Markworth JF, Maddipati KR, Cameron-Smith D. Emerging roles of pro-resolving lipid mediators in immunological and adaptive responses to exercise-induced muscle injury. Exerc Immunol Rev. 2016;22:110–34. 26853678

[29] Bhattacharya A, Hamilton R, Jernigan A, Zhang Y, Sabia M, Rahman MM, Genetic ablation of 12/15-lipoxygenase but not 5-lipoxygenase protects against denervation-induced muscle atrophy. Free Radic Biol Med. 2014;67:30–40. 24121057 10.1016/j.freeradbiomed.2013.10.002

[30] Sun D, Funk CD. Disruption of 12/15-lipoxygenase expression in peritoneal macrophages. Enhanced utilization of the 5-lipoxygenase pathway and diminished oxidation of low density lipoprotein. J Biol Chem. 1996;271(39):24055–62. 8798642

[31] Mayeuf-Louchart A, Hardy D, Thorel Q, Roux P, Gueniot L, Briand D, MuscleJ: a high-content analysis method to study skeletal muscle with a new Fiji tool. Skelet Muscle. 2018;8(1):25. 30081940 10.1186/s13395-018-0171-0 PMC6091189

[32] Hindi L, McMillan JD, Afroze D, Hindi SM, Kumar A. Isolation, Culturing, and Differentiation of Primary Myoblasts from Skeletal Muscle of Adult Mice. Bio Protoc. 2017;7(9). 10.21769/BioProtoc.2248 PMC5515488 28730161

[33] Markworth JF, Cameron-Smith D. Arachidonic acid supplementation enhances in vitro skeletal muscle cell growth via a COX-2-dependent pathway. Am J Physiol Cell Physiol. 2013;304(1):C56–67. 23076795 10.1152/ajpcell.00038.2012

[34] Markworth JF, Sugg KB, Sarver DC, Maddipati KR, Brooks SV. Local shifts in inflammatory and resolving lipid mediators in response to tendon overuse. FASEB J. 2021;35(6):e21655. 34042218 10.1096/fj.202100078R PMC9527947

[35] Pang Z, Lu Y, Zhou G, Hui F, Xu L, Viau C, MetaboAnalyst 6.0: towards a unified platform for metabolomics data processing, analysis and interpretation. Nucleic Acids Res. 2024;52(W1):W398–W406. 38587201 10.1093/nar/gkae253 PMC11223798

[36] Burkholder TJ, Fingado B, Baron S, Lieber RL. Relationship between muscle fiber types and sizes and muscle architectural properties in the mouse hindlimb. J Morphol. 1994;221(2):177–90. 7932768 10.1002/jmor.1052210207

[37] Mendez J, Keys A. Density and Composition of Mammalian Muscle. Metabolism. 1960;9:184–8.

[38] Lee BR, Paing MH, Sharma-Walia N. Cyclopentenone Prostaglandins: Biologically Active Lipid Mediators Targeting Inflammation. Front Physiol. 2021;12:640374. 34335286 10.3389/fphys.2021.640374 PMC8320392

[39] Kim SN, Akindehin S, Kwon HJ, Son YH, Saha A, Jung YS, Anti-inflammatory role of 15-lipoxygenase contributes to the maintenance of skin integrity in mice. Sci Rep. 2018;8(1):8856. 29891910 10.1038/s41598-018-27221-7 PMC5995961

[40] Dioszeghy V, Rosas M, Maskrey BH, Colmont C, Topley N, Chaitidis P, 12/15-Lipoxygenase regulates the inflammatory response to bacterial products in vivo. J Immunol. 2008;181(9):6514–24. 18941242 10.4049/jimmunol.181.9.6514

[41] Wang Y, Welc SS, Wehling-Henricks M, Tidball JG. Myeloid cell-derived tumor necrosis factor-alpha promotes sarcopenia and regulates muscle cell fusion with aging muscle fibers. Aging Cell. 2018;17(6):e12828. 30256507 10.1111/acel.12828 PMC6260911

[42] Wang Y, Wehling-Henricks M, Welc SS, Fisher AL, Zuo Q, Tidball JG. Aging of the immune system causes reductions in muscle stem cell populations, promotes their shift to a fibrogenic phenotype, and modulates sarcopenia. FASEB J. 2019;33(1):1415–27. 30130434 10.1096/fj.201800973R PMC6355087

[43] Wang Y, Wehling-Henricks M, Samengo G, Tidball JG. Increases of M2a macrophages and fibrosis in aging muscle are influenced by bone marrow aging and negatively regulated by muscle-derived nitric oxide. Aging Cell. 2015;14(4):678–88. 26009878 10.1111/acel.12350 PMC4531081

[44] Rieger L, Molina T, Fabre P, Greffard K, Pellerito O, Dort J, Transcriptomic and lipidomic profiling reveals distinct bioactive lipid signatures in slow and fast muscles and highlights the role of resolvin-D2 in fiber type determination during myogenesis. FASEB J. 2024;38(24):e70250. 39698915 10.1096/fj.202401747R PMC11656512

[45] Penner AL, Waytt V, Winter T, Leng S, Duhamel TA, Aukema HM. Oxylipin profiles and levels vary by skeletal muscle type, dietary fat and sex in young rats. Appl Physiol Nutr Metab. 2021;46(11):1378–88. 34115947 10.1139/apnm-2021-0161

[46] Miyoshi M, Usami M, Nishiyama Y, Kai M, Suzuki A, Maeshige N, Soleus muscle contains a higher concentration of lipid metabolites than extensor digitorum longus in rats with lipopolysaccharide-induced acute muscle atrophy. Clin Nutr ESPEN. 2023;57:48–57. 37739695 10.1016/j.clnesp.2023.06.011

[47] Kronke G, Katzenbeisser J, Uderhardt S, Zaiss MM, Scholtysek C, Schabbauer G, 12/15-lipoxygenase counteracts inflammation and tissue damage in arthritis. J Immunol. 2009;183(5):3383–9. 19675173 10.4049/jimmunol.0900327

[48] Jackson CD, Hilliard KA, Brown CR. 12/15-lipoxygenase activity promotes efficient inflammation resolution in a murine model of Lyme arthritis. Front Immunol. 2023;14:1144172. 37143678 10.3389/fimmu.2023.1144172 PMC10151577

[49] Queck A, Fink AF, Sirait-Fischer E, Ruschenbaum S, Thomas D, Snodgrass RG, Alox12/15 Deficiency Exacerbates, While Lipoxin A(4) Ameliorates Hepatic Inflammation in Murine Alcoholic Hepatitis. Front Immunol. 2020;11:1447. 32760397 10.3389/fimmu.2020.01447 PMC7371948

[50] Leedom AJ, Sullivan AB, Dong B, Lau D, Gronert K. Endogenous LXA4 circuits are determinants of pathological angiogenesis in response to chronic injury. Am J Pathol. 2010;176(1):74–84. 20008149 10.2353/ajpath.2010.090678 PMC2797871

[51] Miyata J, Yokokura Y, Moro K, Arai H, Fukunaga K, Arita M. 12/15-Lipoxygenase Regulates IL-33-Induced Eosinophilic Airway Inflammation in Mice. Front Immunol. 2021;12:687192. 34093589 10.3389/fimmu.2021.687192 PMC8170304

[52] Heydeck D, Kakularam KR, Labuz D, Machelska H, Rohwer N, Weylandt K, Transgenic mice overexpressing human ALOX15 under the control of the aP2 promoter are partly protected in the complete Freund’s adjuvant-induced paw inflammation model. Inflamm Res. 2023;72(8):1649–64. 37498393 10.1007/s00011-023-01770-8 PMC10499711

[53] Halade GV, Kain V, Ingle KA, Prabhu SD. Interaction of 12/15-lipoxygenase with fatty acids alters the leukocyte kinetics leading to improved postmyocardial infarction healing. Am J Physiol Heart Circ Physiol. 2017;313(1):H89–H102. 28411230 10.1152/ajpheart.00040.2017 PMC5538863

[54] Kain V, Ingle KA, Kabarowski J, Barnes S, Limdi NA, Prabhu SD, Genetic deletion of 12/15 lipoxygenase promotes effective resolution of inflammation following myocardial infarction. J Mol Cell Cardiol. 2018;118:70–80. 29526491 10.1016/j.yjmcc.2018.03.004 PMC5940552

[55] Kain V, Ingle KA, Rajasekaran NS, Halade GV. Activation of EP4 receptor limits transition of acute to chronic heart failure in lipoxygenase deficient mice. Theranostics. 2021;11(6):2742–54. 33456570 10.7150/thno.51183 PMC7806484

[56] Kain V, Grilo GA, Upadhyay G, Nadler JL, Serhan CN, Halade GV. Macrophage-specific lipoxygenase deletion amplify cardiac repair activating Treg cells in chronic heart failure. J Leukoc Biol. 2024;116(4):864–75. 38785336 10.1093/jleuko/qiae113 PMC11444306

[57] Gaberel T, Gakuba C, Zheng Y, Lepine M, Lo EH, van Leyen K. Impact of 12/15-Lipoxygenase on Brain Injury After Subarachnoid Hemorrhage. Stroke. 2019;50(2):520–3. 30602353 10.1161/STROKEAHA.118.022325 PMC6349484

[58] Zarbock A, Distasi MR, Smith E, Sanders JM, Kronke G, Harry BL, Improved survival and reduced vascular permeability by eliminating or blocking 12/15-lipoxygenase in mouse models of acute lung injury (ALI). J Immunol. 2009;183(7):4715–22. 19752233 10.4049/jimmunol.0802592 PMC2753988

[59] Rossaint J, Nadler JL, Ley K, Zarbock A. Eliminating or blocking 12/15-lipoxygenase reduces neutrophil recruitment in mouse models of acute lung injury. Crit Care. 2012;16(5):R166. 22973824 10.1186/cc11518 PMC3682261

[60] Wen Y, Gu J, Vandenhoff GE, Liu X, Nadler JL. Role of 12/15-lipoxygenase in the expression of MCP-1 in mouse macrophages. Am J Physiol Heart Circ Physiol. 2008;294(4):H1933–8. 18296557 10.1152/ajpheart.00260.2007

[61] Kayama Y, Minamino T, Toko H, Sakamoto M, Shimizu I, Takahashi H, Cardiac 12/15 lipoxygenase-induced inflammation is involved in heart failure. J Exp Med. 2009;206(7):1565–74. 19546247 10.1084/jem.20082596 PMC2715088

[62] Shireman PK, Contreras-Shannon V, Ochoa O, Karia BP, Michalek JE, McManus LM. MCP-1 deficiency causes altered inflammation with impaired skeletal muscle regeneration. J Leukoc Biol. 2007;81(3):775–85. 17135576 10.1189/jlb.0506356

[63] Martinez CO, McHale MJ, Wells JT, Ochoa O, Michalek JE, McManus LM, Regulation of skeletal muscle regeneration by CCR2-activating chemokines is directly related to macrophage recruitment. Am J Physiol Regul Integr Comp Physiol. 2010;299(3):R832–42. 20631294 10.1152/ajpregu.00797.2009 PMC2944434

[64] Zhang MJ, Sansbury BE, Hellmann J, Baker JF, Guo L, Parmer CM, Resolvin D2 Enhances Postischemic Revascularization While Resolving Inflammation. Circulation. 2016;134(9):666–80. 27507404 10.1161/CIRCULATIONAHA.116.021894 PMC5214591

[65] Dort J, Orfi Z, Fabre P, Molina T, Conte TC, Greffard K, Resolvin-D2 targets myogenic cells and improves muscle regeneration in Duchenne muscular dystrophy. Nat Commun. 2021;12(1):6264. 34716330 10.1038/s41467-021-26516-0 PMC8556273

[66] Dort J, Orfi Z, Fiscaletti M, Campeau PM, Dumont NA. Gpr18 agonist dampens inflammation, enhances myogenesis, and restores muscle function in models of Duchenne muscular dystrophy. Front Cell Dev Biol. 2023;11:1187253. 37645248 10.3389/fcell.2023.1187253 PMC10461444

[67] Perazza LR, Gower AC, Brown-Borg HM, Pajevic PD, Thompson LV. Protectin DX as a therapeutic strategy against frailty in mice. Geroscience. 2023;45(4):2601–27. 37059838 10.1007/s11357-023-00789-3 PMC10651819

[68] Baker LA, Martin NRW, Kimber MC, Pritchard GJ, Lindley MR, Lewis MP. Resolvin E1 (R(v) E(1)) attenuates LPS induced inflammation and subsequent atrophy in C2C12 myotubes. J Cell Biochem. 2018;119(7):6094–103. 29574938 10.1002/jcb.26807

[69] Zong H, Li X, Lin H, Hou C, Ma F. Lipoxin A4 pretreatment mitigates skeletal muscle ischemia-reperfusion injury in rats. Am J Transl Res. 2017;9(3):1139–50. 28386340 PMC5376005

[70] Serhan CN, Sheppard KA. Lipoxin formation during human neutrophil-platelet interactions. Evidence for the transformation of leukotriene A4 by platelet 12-lipoxygenase in vitro. J Clin Invest. 1990;85(3):772–80. 2155925 10.1172/JCI114503 PMC296494

[71] Poeckel D, Zemski Berry KA, Murphy RC, Funk CD. Dual 12/15- and 5-lipoxygenase deficiency in macrophages alters arachidonic acid metabolism and attenuates peritonitis and atherosclerosis in ApoE knockout mice. J Biol Chem. 2009;284(31):21077–89. 19509298 10.1074/jbc.M109.000901 PMC2742872

[72] Titos E, Rius B, Gonzalez-Periz A, Lopez-Vicario C, Moran-Salvador E, Martinez-Clemente M, Resolvin D1 and its precursor docosahexaenoic acid promote resolution of adipose tissue inflammation by eliciting macrophage polarization toward an M2-like phenotype. J Immunol. 2011;187(10):5408–18. 22013115 10.4049/jimmunol.1100225

[73] Dalli J, Serhan CN. Specific lipid mediator signatures of human phagocytes: microparticles stimulate macrophage efferocytosis and pro-resolving mediators. Blood. 2012;120(15):e60–72. 22904297 10.1182/blood-2012-04-423525 PMC3471524

[74] Werz O, Gerstmeier J, Libreros S, De la Rosa X, Werner M, Norris PC, Human macrophages differentially produce specific resolvin or leukotriene signals that depend on bacterial pathogenicity. Nat Commun. 2018;9(1):59. 29302056 10.1038/s41467-017-02538-5 PMC5754355

[75] Miller YI, Chang MK, Funk CD, Feramisco JR, Witztum JL. 12/15-lipoxygenase translocation enhances site-specific actin polymerization in macrophages phagocytosing apoptotic cells. J Biol Chem. 2001;276(22):19431–9. 11278875 10.1074/jbc.M011276200

[76] Uderhardt S, Herrmann M, Oskolkova OV, Aschermann S, Bicker W, Ipseiz N, 12/15-lipoxygenase orchestrates the clearance of apoptotic cells and maintains immunologic tolerance. Immunity. 2012;36(5):834–46. 22503541 10.1016/j.immuni.2012.03.010

[77] Huang JT, Welch JS, Ricote M, Binder CJ, Willson TM, Kelly C, Interleukin-4-dependent production of PPAR-gamma ligands in macrophages by 12/15-lipoxygenase. Nature. 1999;400(6742):378–82. 10432118 10.1038/22572

[78] Ogawa M, Ishihara T, Isobe Y, Kato T, Kuba K, Imai Y, Eosinophils promote corneal wound healing via the 12/15-lipoxygenase pathway. FASEB J. 2020;34(9):12492–501. 32721046 10.1096/fj.202000483R

[79] Gronert K, Maheshwari N, Khan N, Hassan IR, Dunn M, Laniado Schwartzman M. A role for the mouse 12/15-lipoxygenase pathway in promoting epithelial wound healing and host defense. J Biol Chem. 2005;280(15):15267–78. 15708862 10.1074/jbc.M410638200

[80] Cai W, Liu L, Shi X, Liu Y, Wang J, Fang X, Alox15/15-HpETE Aggravates Myocardial Ischemia-Reperfusion Injury by Promoting Cardiomyocyte Ferroptosis. Circulation. 2023;147(19):1444–60. 36987924 10.1161/CIRCULATIONAHA.122.060257

[81] Pizza FX, Peterson JM, Baas JH, Koh TJ. Neutrophils contribute to muscle injury and impair its resolution after lengthening contractions in mice. J Physiol. 2005;562(Pt 3):899–913. 15550464 10.1113/jphysiol.2004.073965 PMC1665528

[82] Arnold L, Henry A, Poron F, Baba-Amer Y, van Rooijen N, Plonquet A, Inflammatory monocytes recruited after skeletal muscle injury switch into antiinflammatory macrophages to support myogenesis. J Exp Med. 2007;204(5):1057–69. 17485518 10.1084/jem.20070075 PMC2118577

[83] McLennan IS. Degenerating and regenerating skeletal muscles contain several subpopulations of macrophages with distinct spatial and temporal distributions. J Anat. 1996;188 (Pt 1)(Pt 1):17–28. 8655404 PMC1167629

[84] Nie M, Liu J, Yang Q, Seok HY, Hu X, Deng ZL, MicroRNA-155 facilitates skeletal muscle regeneration by balancing pro- and anti-inflammatory macrophages. Cell Death Dis. 2016;7(6):e2261. 27277683 10.1038/cddis.2016.165 PMC5143393

[85] Wang H, Melton DW, Porter L, Sarwar ZU, McManus LM, Shireman PK. Altered macrophage phenotype transition impairs skeletal muscle regeneration. Am J Pathol. 2014;184(4):1167–84. 24525152 10.1016/j.ajpath.2013.12.020 PMC3969996

[86] Akahori H, Karmali V, Polavarapu R, Lyle AN, Weiss D, Shin E, CD163 interacts with TWEAK to regulate tissue regeneration after ischaemic injury. Nat Commun. 2015;6:7792. 26242746 10.1038/ncomms8792 PMC4918310

[87] Nawaz A, Bilal M, Fujisaka S, Kado T, Aslam MR, Ahmed S, Depletion of CD206(+) M2-like macrophages induces fibro-adipogenic progenitors activation and muscle regeneration. Nat Commun. 2022;13(1):7058. 36411280 10.1038/s41467-022-34191-y PMC9678897

[88] Ito H, Ueda H, Iwamoto I, Inaguma Y, Takizawa T, Asano T, Nordihydroguaiaretic acid (NDGA) blocks the differentiation of C2C12 myoblast cells. J Cell Physiol. 2005;202(3):874–9. 15389564 10.1002/jcp.20177

[89] Lehmann C, Homann J, Ball AK, Blocher R, Kleinschmidt TK, Basavarajappa D, Lipoxin and resolvin biosynthesis is dependent on 5-lipoxygenase activating protein. FASEB J. 2015;29(12):5029–43. 26289316 10.1096/fj.15-275487

[90] Mainka M, George S, Angioni C, Ebert R, Goebel T, Kampschulte N, On the biosynthesis of specialized pro-resolving mediators in human neutrophils and the influence of cell integrity. Biochim Biophys Acta Mol Cell Biol Lipids. 2022;1867(3):159093. 34942381 10.1016/j.bbalip.2021.159093

[91] Dahlke P, Peltner LK, Jordan PM, Werz O. Differential impact of 5-lipoxygenase-activating protein antagonists on the biosynthesis of leukotrienes and of specialized pro-resolving mediators. Front Pharmacol. 2023;14:1219160. 37680719 10.3389/fphar.2023.1219160 PMC10481534

[92] Smith HJ, Lorite MJ, Tisdale MJ. Effect of a cancer cachectic factor on protein synthesis/degradation in murine C2C12 myoblasts: modulation by eicosapentaenoic acid. Cancer Res. 1999;59(21):5507–13. 10554027

[93] Whitehouse AS, Khal J, Tisdale MJ. Induction of protein catabolism in myotubes by 15(S)-hydroxyeicosatetraenoic acid through increased expression of the ubiquitin-proteasome pathway. Br J Cancer. 2003;89(4):737–45. 12915888 10.1038/sj.bjc.6601184 PMC2376908

[94] Wyke SM, Khal J, Tisdale MJ. Signalling pathways in the induction of proteasome expression by proteolysis-inducing factor in murine myotubes. Cell Signal. 2005;17(1):67–75. 15451026 10.1016/j.cellsig.2004.05.015

[95] Russell ST, Eley H, Tisdale MJ. Role of reactive oxygen species in protein degradation in murine myotubes induced by proteolysis-inducing factor and angiotensin II. Cell Signal. 2007;19(8):1797–806. 17532611 10.1016/j.cellsig.2007.04.003

[96] Leng X, Jiang H. Effects of arachidonic acid and its major prostaglandin derivatives on bovine myoblast proliferation, differentiation, and fusion. Domest Anim Endocrinol. 2019;67:28–36. 30677541 10.1016/j.domaniend.2018.12.006

[97] Briolay A, Jaafar R, Nemoz G, Bessueille L. Myogenic differentiation and lipid-raft composition of L6 skeletal muscle cells are modulated by PUFAs. Biochim Biophys Acta. 2013;1828(2):602–13. 23079583 10.1016/j.bbamem.2012.10.006

[98] Risha MA, Siengdee P, Dannenberger D, Wimmers K, Ponsuksili S. PUFA Treatment Affects C2C12 Myocyte Differentiation, Myogenesis Related Genes and Energy Metabolism. Genes (Basel). 2021;12(2). 10.3390/genes12020192 PMC7910949 33525599

[99] Lee JH, Tachibana H, Morinaga Y, Fujimura Y, Yamada K. Modulation of proliferation and differentiation of C2C12 skeletal muscle cells by fatty acids. Life Sci. 2009;84(13-14):415–20. 19302823 10.1016/j.lfs.2009.01.004

[100] Mitra A, Shanavas S, Chaudhury D, Bose B, Das UN, Shenoy PS. Mitigation of chronic glucotoxicity-mediated skeletal muscle atrophy by arachidonic acid. Life Sci. 2023;333:122141. 37797688 10.1016/j.lfs.2023.122141

[101] Tachtsis B, Whitfield J, Hawley JA, Hoffman NJ. Omega-3 Polyunsaturated Fatty Acids Mitigate Palmitate-Induced Impairments in Skeletal Muscle Cell Viability and Differentiation. Front Physiol. 2020;11:563. 32581844 10.3389/fphys.2020.00563 PMC7283920

[102] Magee P, Pearson S, Allen J. The omega-3 fatty acid, eicosapentaenoic acid (EPA), prevents the damaging effects of tumour necrosis factor (TNF)-alpha during murine skeletal muscle cell differentiation. Lipids Health Dis. 2008;7:24. 18638380 10.1186/1476-511X-7-24 PMC2500009

[103] Magee P, Pearson S, Whittingham-Dowd J, Allen J. PPARgamma as a molecular target of EPA anti-inflammatory activity during TNF-alpha-impaired skeletal muscle cell differentiation. J Nutr Biochem. 2012;23(11):1440–8. 22305406 10.1016/j.jnutbio.2011.09.005

[104] Saini A, Sharples AP, Al-Shanti N, Stewart CE. Omega-3 fatty acid EPA improves regenerative capacity of mouse skeletal muscle cells exposed to saturated fat and inflammation. Biogerontology. 2017;18(1):109–29. 27864687 10.1007/s10522-016-9667-3 PMC5288450

[105] Li C, Cao H, Ren Y, Jia J, Yang G, Jin J, Eicosapentaenoic acid-mediated activation of PGAM2 regulates skeletal muscle growth and development via the PI3K/AKT pathway. Int J Biol Macromol. 2024;268(Pt 2):131547. 38641281 10.1016/j.ijbiomac.2024.131547

[106] Xu J, Liu D, Yin H, Tong H, Li S, Yan Y. Fatty acids promote bovine skeletal muscle satellite cell differentiation by regulating ELOVL3 expression. Cell Tissue Res. 2018;373(2):499–508. 29464364 10.1007/s00441-018-2812-3

[107] Oh H, Cho W, Abd El-Aty AM, Bayram C, Jeong JH, Jung TW. Resolvin D3 improves the impairment of insulin signaling in skeletal muscle and nonalcoholic fatty liver disease through AMPK/autophagy-associated attenuation of ER stress. Biochem Pharmacol. 2022;203:115203. 35948170 10.1016/j.bcp.2022.115203

