# Supplementary material for: Leukocyte-type 12/15-lipoxygenase is essential for timely inflammation-resolution and effective tissue regeneration following skeletal muscle injury
Source: bioRxiv. 2025 May 17:2025.05.13.653766. Preprint. [Version 1] doi: 10.1101/2025.05.13.653766 (PMC12132247; doi:10.1101/2025.05.13.653766)

bioRxiv preprint doi: https://doi.org/10.1101/2025.05.13.653766; this version posted May 17, 2025. The copyright holder for this preprint (which was not certified by peer review) is the author/funder, who has granted bioRxiv a license to display the preprint in perpetuity. It is made available under aCC-BY 4.0 International license.

<u>Supplementary Figure 1:</u> Characterization of the basal skeletal muscle phenotype of 12/15-LOX deficient mice. A: Body weights (g) of wild type (WT) and *Alox15* knockout (*Alox15*$^{-/-}$) mice. **B:** Measurement of *in situ* absolute maximal isometric force (mN) produced by the tibialis anterior (TA) muscle of WT and *Alox15*$^{-/-}$ mice. **C:** Maximal specific isometric contractile force (sP_O), mN/mm²) of WT and *Alox15*$^{-/-}$ mice. **D:** Absolute muscle mass of soleus (SOL), plantaris (PLA), tibialis anterior (TA), gastrocnemius (GAST), and quadriceps (QUAD) muscles from WT and *Alox15*$^{-/-}$ mice. **E:** TA cross-sections from WT and *Alox15*$^{-/-}$ mice were stained with antibodies against myosin heavy chain (MyHC) isoforms I, IIA, and IIB to identify specific muscle fiber types. Type IIx fibers remain unstained (black). Bars show the mean ± SEM of 4-5 mice per group (biological replicates) with dots representing data from each individual mouse. P-values were determined by two-tailed unpaired t-tests. *p<0.05 WT vs. *Alox15*$^{-/-}$ mice. Scale bars are 1000 µm for stitched whole muscle sections and 200 µm for representative fields of view.

<u>Supplementary Figure 2:</u> Effect of 12/15-LOX knockout on local expression of inflammatory cytokines and growth factors following skeletal muscle injury. A-E: Muscle mRNA expression of cytokines/chemokines including TNFα (*Tnf1*) (**A**), IL-1β (*Il1b*) (**B**) IL-6 (*Il6*) (**C**), IL-10 (*Il10*) (**D**), and MCP-1 (*Ccl2*) (**E**) at day 3 (D3), day 5 (D5), and day 14 (D14) following muscle injury induced by intramuscular injection of barium chloride ($BaCL_2$). **F-J:** Muscle mRNA expression of growth factors including IGF-1 (*Igf1*) (**F**), EGF (*Egf*) (**G**), TGF-β (*Tgfb1*) (**H**), arginase 1 (*Arg1*) (**I**), and VEGF (*Vegfa*) (**J**) at D3, D5, and D14 following muscle injury induced by intramuscular injection of $BaCL_2$**.** Bars show the mean ± SEM of 4-5 mice per group (biological replicates with dots representing data from each individual mouse. P-values were determined by two-way ANOVA followed by Holm-Šídák post-hoc tests. *p<0.05 vs. D0 and #p<0.05 for WT vs. *Alox15*$^{-/-}$ mice.

<u>Supplementary Figure 3:</u> Metabolipidomic profiling reveals a local imbalance of pro-inflammatory vs. anti-inflammatory/pro-resolving lipid mediators following muscle injury. A-F: Intramuscular concentrations of major metabolites of the COX pathway (**A**), 5-LOX pathway (**B**), 12-LOX pathway (**C**), 15-LOX pathway (**D**), CYP pathway (**E**), and downstream bioactive SPMs (**F**) detected at day 3 (D3), day 5 (D5), and day 14 (D14) following muscle injury induced by intramuscular injection of $BaCl_2$. Bars show the mean ± SEM of 4-5 mice per group (biological replicates) with dots representing data from each individual mouse. P-values were determined by two-way ANOVA followed by Holm-Šídák post-hoc tests. *p<0.05 vs. D0 and #p<0.05 for WT vs. *Alox15*$^{-/-}$ mice.

<u>Supplementary Figure 4:</u> Effect of 12/15-LOX knockout on expression of inflammation related genes in bone marrow derived macrophages. Bone marrow cells were cultured for 7 days in the present of M-CSF to obtain adherent bone marrow-derived macrophages (BMMs). BMMs were maintained in serum free media lacking M-CSF for 24 h to obtain naïve M0 BMM, or polarized to a M1 or M2 phenotype by 24 h treatment with LPS (100 ng/mL) + INF-γ (20 ng/mL) or IL-4 (20 ng/mL), respectively. Expression of mRNA encoding F4/80 (*Adgre1*) (**A**), MCP-1 (*Ccl2*) (**B**), TNFα (*Tnf*) (**C**), M-CSF (*Csf1*) (**D**), IL-10 (*Il10*) (**E**), GM-CSF (*Csf2*) (**F**), CD68 (*Cd68*) (**G**), and FLAP (*Alox5ap*) (**H**) in BMMs obtained from wild type (WT) and 12/15-LOX deficient (*Alox15*$^{-/-}$)

bioRxiv preprint doi: https://doi.org/10.1101/2025.05.13.653766; this version posted May 17, 2025. The copyright holder for this preprint (which was not certified by peer review) is the author/funder, who has granted bioRxiv a license to display the preprint in perpetuity. It is made available under aCC-BY 4.0 International license.

mice. Gene expression was measured via RT-qPCR. P-values were determined by two-way ANOVA with Holm-Šídák post-hoc tests. *p<0.05 vs. M0, &p<0.05 vs. M1, and #p<0.05 for WT vs. *Alox15$^{-/-}$* cells.

__Supplementary Figure 5:__ **Bone marrow-derived macrophages from *Alox15$^{-/-}$* mice are deficient in 12/15-LOX-derived lipid mediators under M2-polarizing conditions. A-F:** Extracellular concentrations of major metabolites of the cyclooxygenase (COX) pathway (**A**), 5-lipoxygenase (5-LOX) pathway (**B**), 12-lipoxygenase (12-LOX) pathway (**C**), 15-lipoxygenase (15-LOX) pathway (**D**), cytochrome p450 (CYP) pathway (**E**), and downstream bioactive specialized pro-resolving mediators (SPMs) (**F**) in serum free conditioned cell culture media samples obtained from M0, M1, or M2 bone-marrow-derived macrophage (BMM) cultures from wild type (WT) and 12/15-LOX deficient (*Alox15$^{-/-}$*) mice. Bars show the mean ± SEM of BMMs obtained from n=5 mice per group (biological replicates) with dots representing BMMs from each individual mouse. P-values were determined by two-way ANOVA with Holm-Šídák post-hoc tests. *p<0.05 vs. M0, &p<0.05 vs. M1, and #p<0.05 for WT vs. *Alox15$^{-/-}$* cells.

__Supplementary Figure 6:__ **Pharmacological inhibition of 15-LOX-1 and FLAP markedly suppresses *in vitro* myogenesis.** Murine C2C12 myoblasts were grown to confluence and then induced to undergo myogenic differentiation in the presence of various doses of pharmacological LOX enzyme inhibitors. (**A**): Pan LOX inhibitors including eicosatetraynoic acid (ETYA), nordihydroguaiaretic acid (NDGA), and baicalein. (**B**): 5-LOX specific inhibitors including MK886, malotilate, and zileuton. (**C**): 12-LOX specific inhibitors including CAY10698 and ML355. (**D**): 15-LOX-1 specific inhibitors including BLX3887, 9c(i472), PD146176, ML351, and ThioLox. Following 72 h of myogenic differentiation resulting myotube cultures were fixed in 4% PFA and stained with primary antibodies against sarcomeric myosin heavy chain (MyHC, green), myogenin (MyoG, red). Cell nuclei were counterstained with DAPI (blue). Results are representative of 3 independent experiments. Scale bars are 200 µm.

__Supplementary Figure 7:__ **Myogenic progenitor cells produce specialized pro-resolving mediators (SPMs) via a 12/15-LOX dependent pathway.** Primary mouse myoblasts were induced to differentiate for 72 h in culture media supplemented with vehicle control (0.02% ethanol), a 25 µM dose of individual pure long chain polyunsaturated fatty acids (LC-PUFAs) including arachidonic acid (ARA), eicosapentaenoic acid (EPA), docosapentaenoic acid (DPA), docosahexaenoic acid (DHA), or an equimolar mixture of ARA, EPA, DPA, and DHA (6.25 µM each). Extracellular concentrations of major metabolites of the COX pathway (**A**), 5-LOX pathway (**B**), 12-LOX pathway (**C**), 15-LOX pathway (**D**), CYP pathway (**E**), and downstream bioactive SPMs (**F**) as detected by LC-MS/MS analysis of conditioned culture media samples obtained from differentiating wild type (WT) and *Alox15$^{-/-}$* myoblasts. Bars show the mean ± SEM of primary myoblasts obtained from n=4 mice per group. P-values were determined by two-way ANOVA followed by Holm-Šídák post-hoc tests. *p<0.05 vs. vehicle and #p<0.05 for WT vs. *Alox15$^{-/-}$* myotubes.

bioRxiv preprint doi: https://doi.org/10.1101/2025.05.13.653766; this version posted May 17, 2025. The copyright holder for this preprint (which was not certified by peer review) is the author/funder, who has granted bioRxiv a license to display the preprint in perpetuity. It is made available under aCC-BY 4.0 International license.

Supplemental Figure 1

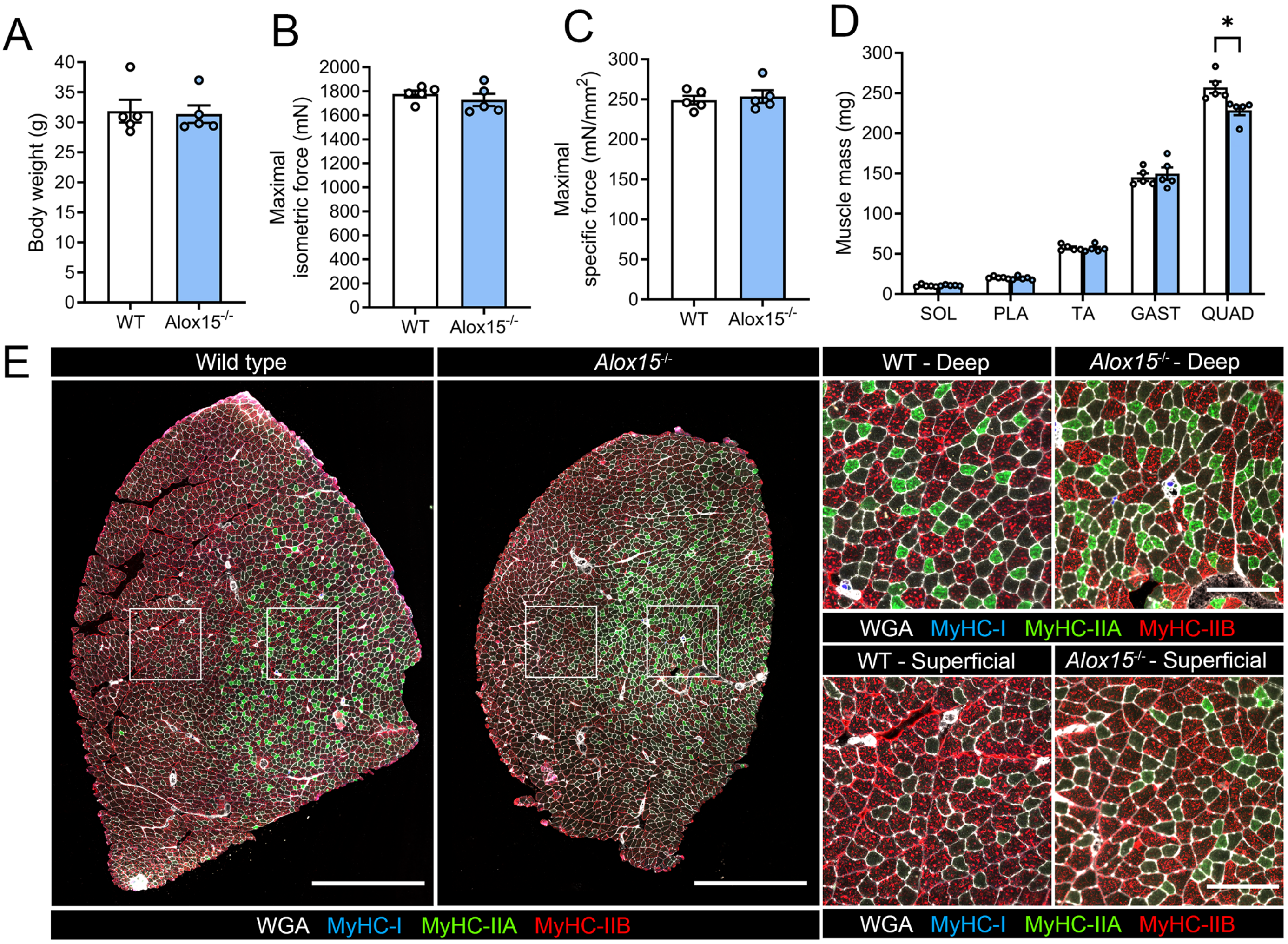

bioRxiv preprint doi: https://doi.org/10.1101/2025.05.13.653766; this version posted May 17, 2025. The copyright holder for this preprint (which was not certified by peer review) is the author/funder, who has granted bioRxiv a license to display the preprint in perpetuity. It is made available under aCC-BY 4.0 International license.

Supplemental Figure 2

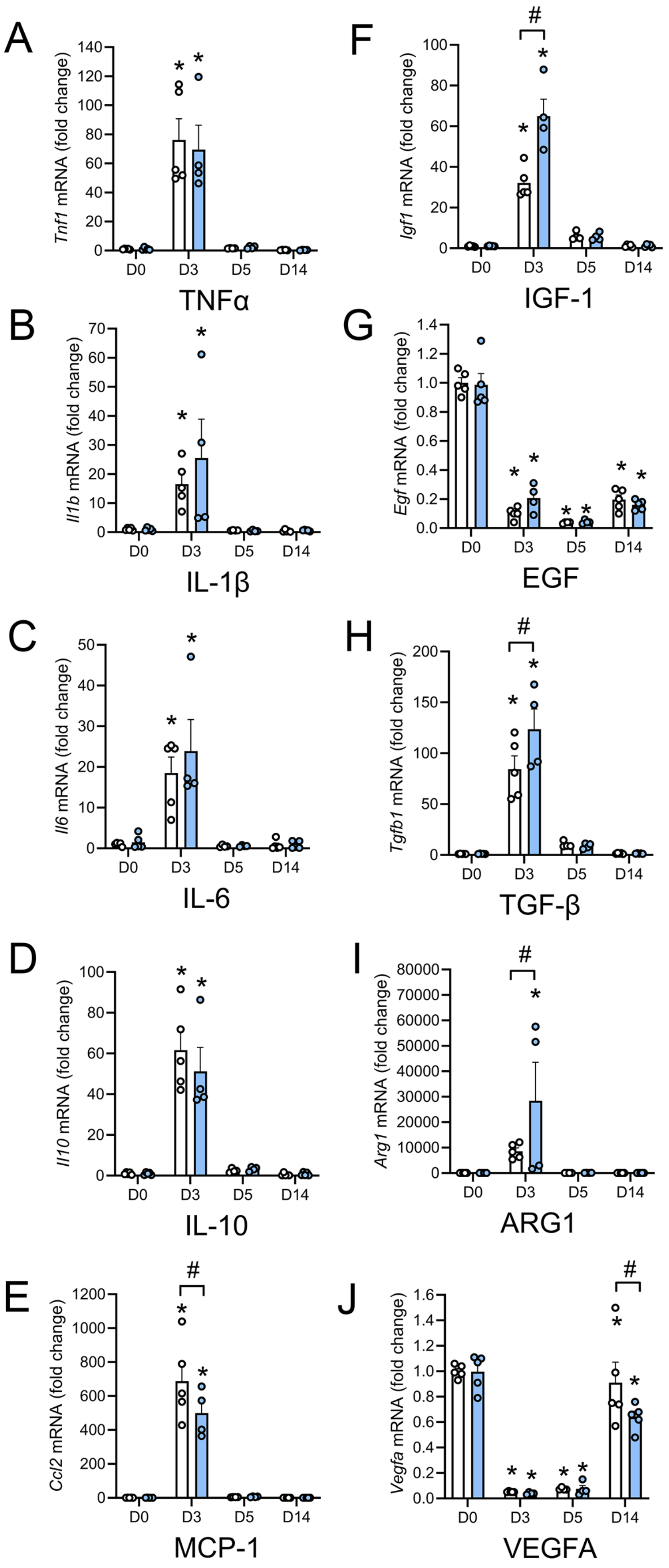

Supplemental Figure 3

bioRxiv preprint doi: https://doi.org/10.1101/2025.05.13.653766; this version posted May 17, 2025. The copyright holder for this preprint (which was not certified by peer review) is the author/funder, who has granted bioRxiv a license to display the preprint in perpetuity. It is made available under aCC-BY 4.0 International license.

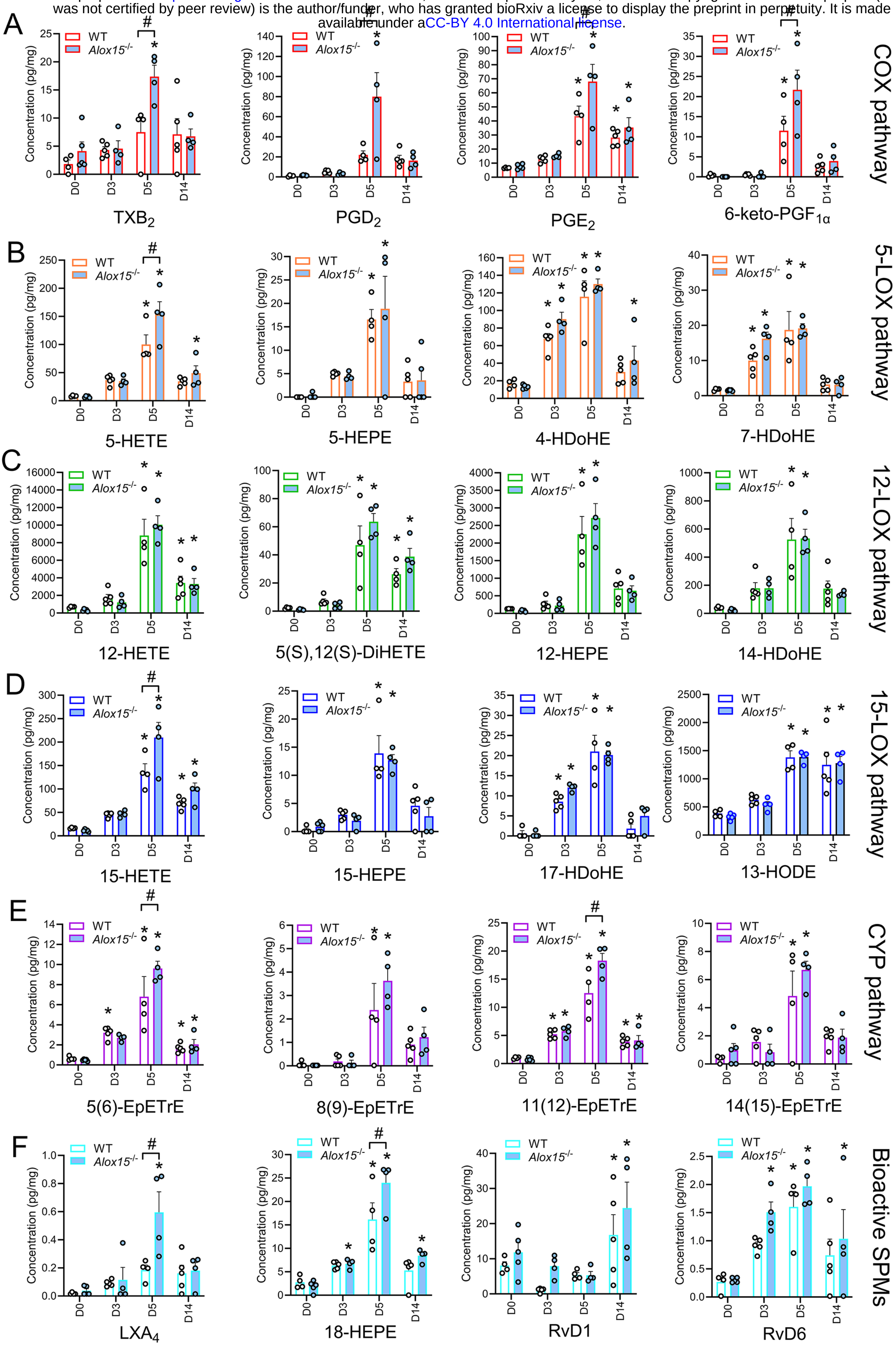

bioRxiv preprint doi: https://doi.org/10.1101/2025.05.13.653766; this version posted May 17, 2025. The copyright holder for this preprint (which was not certified by peer review) is the author/funder, who has granted bioRxiv a license to display the preprint in perpetuity. It is made available under aCC-BY 4.0 International license.

Supplemental Figure 4

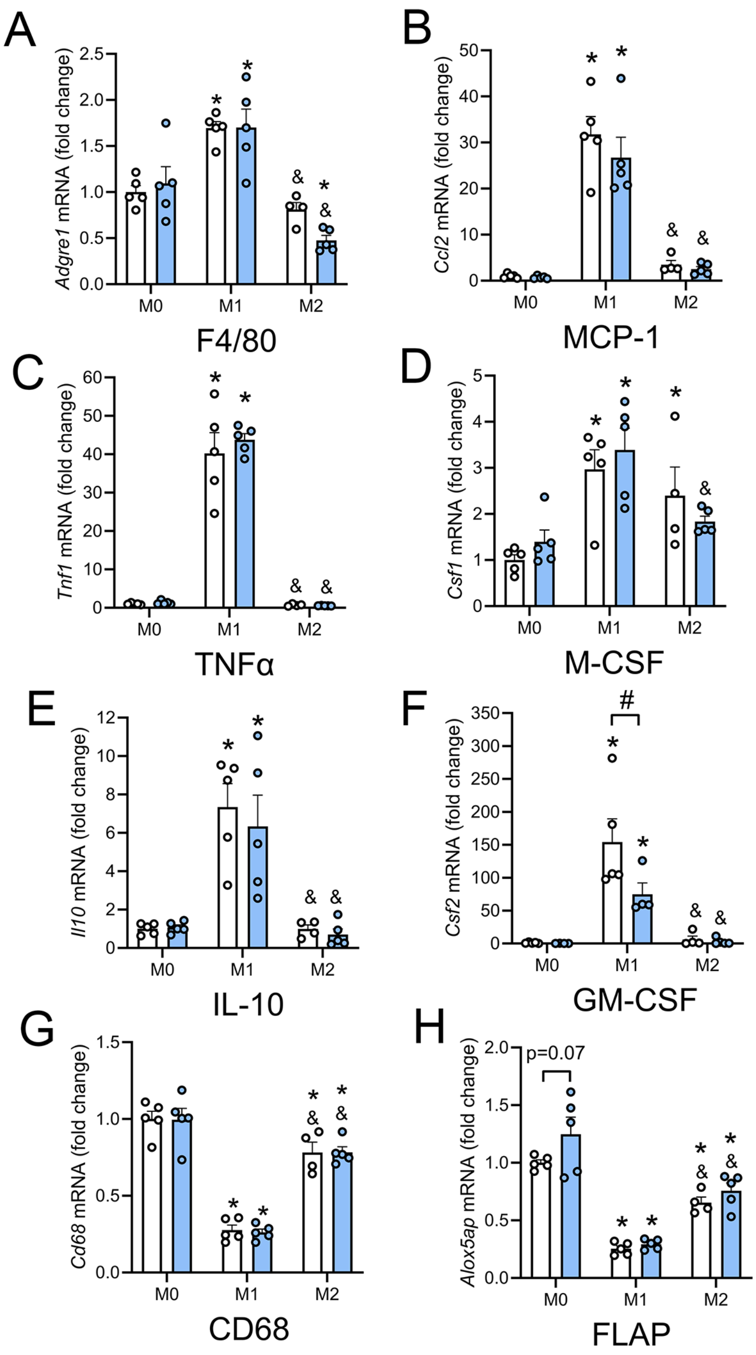

Supplemental Figure 5

bioRxiv preprint doi: https://doi.org/10.1101/2025.05.13.653766; this version posted May 17, 2025. The copyright holder for this preprint (which was not certified by peer review) is the author/funder, who has granted bioRxiv a license to display the preprint in perpetuity. It is made available under aCC-BY 4.0 International license.

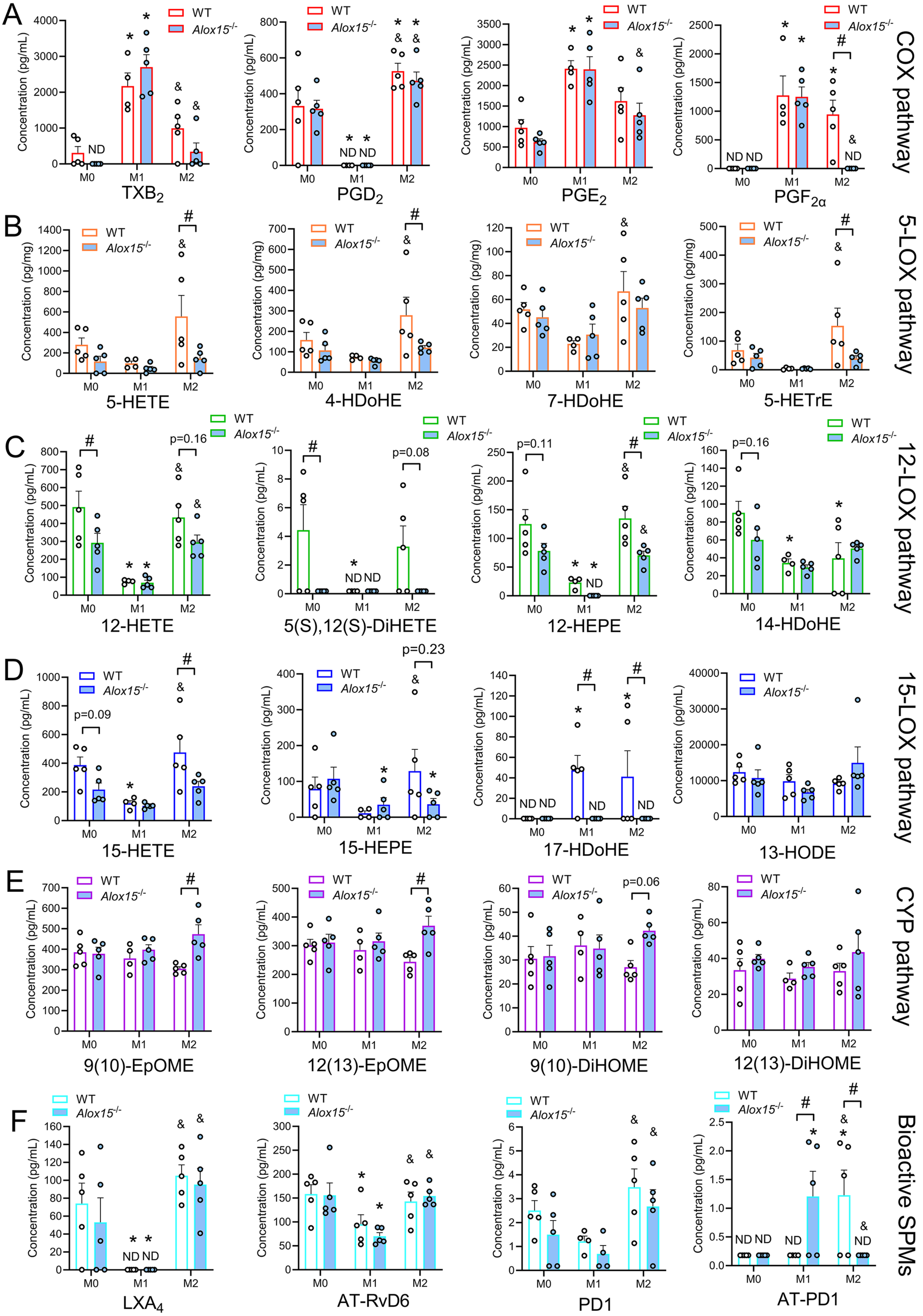

bioRxiv preprint doi: https://doi.org/10.1101/2025.05.13.653766; this version posted May 17, 2025. The copyright holder for this preprint (which was not certified by peer review) is the author/funder, who has granted bioRxiv a license to display the preprint in perpetuity. It is made available under aCC-BY 4.0 International license.

Supplemental Figure 6

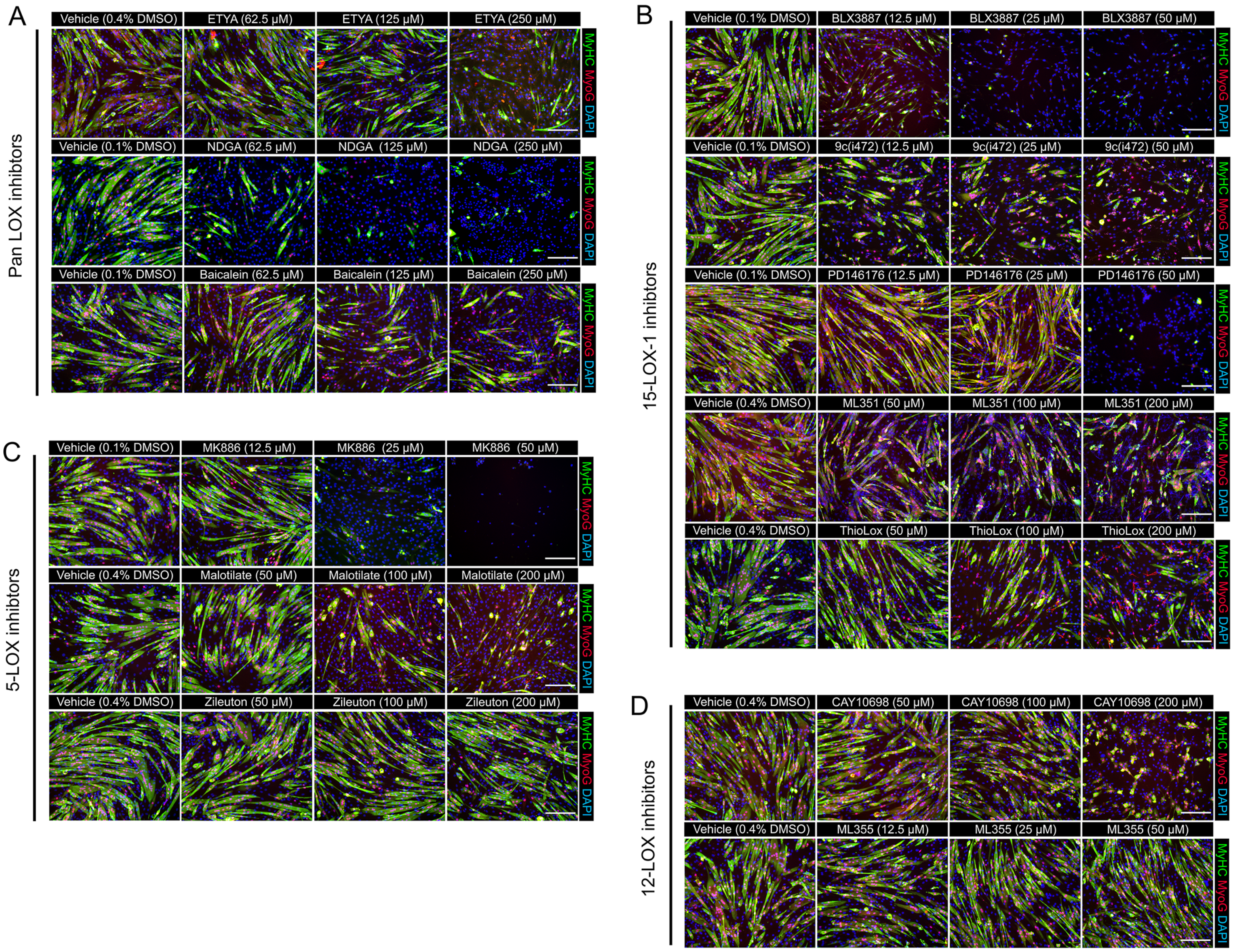

Supplemental Figure 7

bioRxiv preprint doi: https://doi.org/10.1101/2025.05.13.653766; this version posted May 17, 2025. The copyright holder for this preprint (which was not certified by peer review) is the author/funder, who has granted bioRxiv a license to display the preprint in perpetuity. It is made available under aCC-BY 4.0 International license.

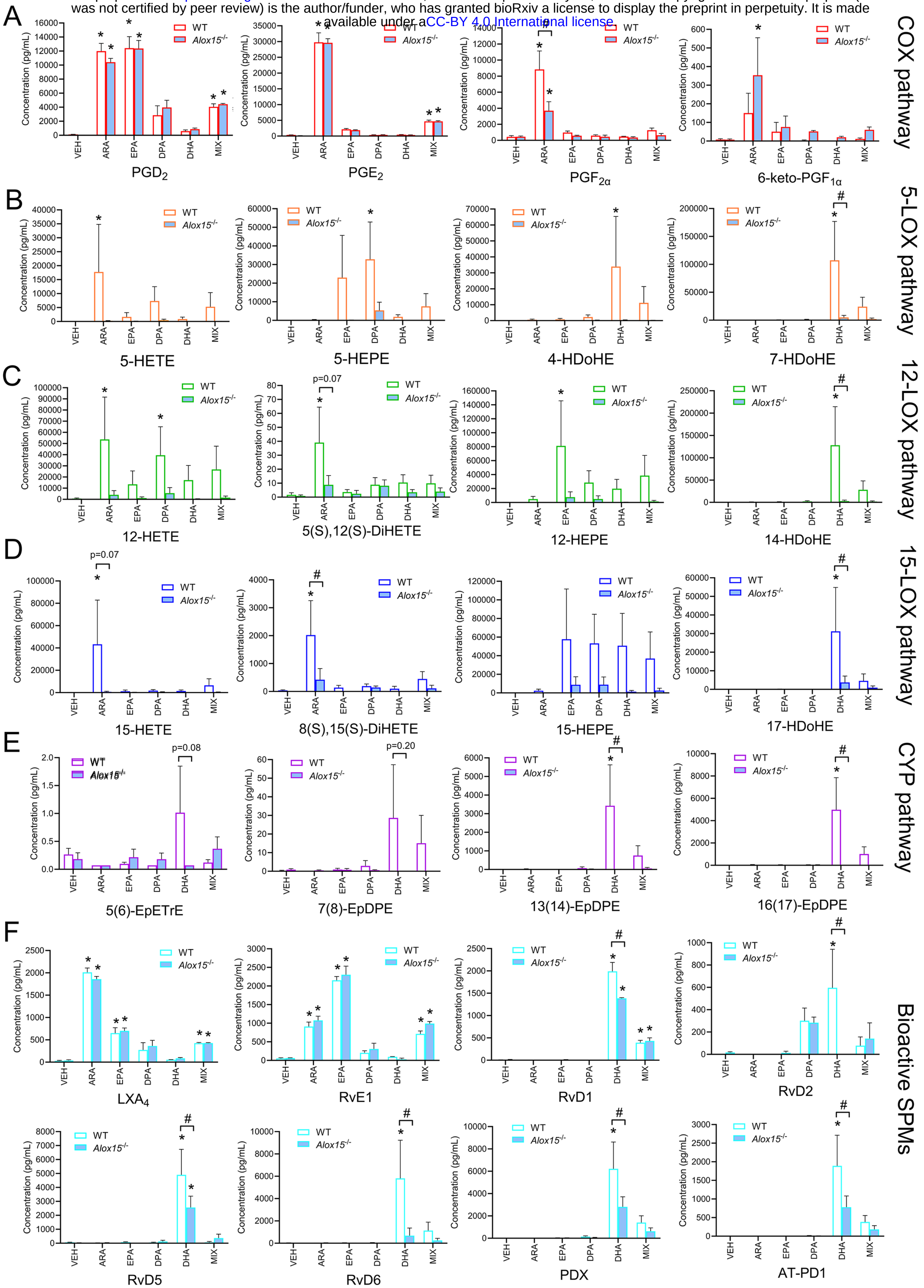

Supplement: 9 [file NIHPP2025.05.13.653766v1-supplement-9.pdf]
